# Comparative Analysis of Methods for Improving the Classification Accuracy of Morphologically Similar Pollen Grain Classes on the CPD-1 Dataset

**DOI:** 10.3390/s26206422

**Published:** 2026-10-11

**Authors:** Abdinabi Mukhamadiyev, Rustam Safarov, Isroil Jumanov, Sunatillo Kholmonov, Toshtemir Khujakulov, Doston Khasanov, Ergashevich Halimjon Khujamatov, Heung Seok Jeon, Cheolwon Lee

**Affiliations:** 1 Department of Computer Engineering, Gachon University, Sujeong-gu, Seongnam-si 13120, Republic of Korea; mukhamadiyev@gachon.ac.kr; 2 Department of Systematic and Practical Programming, Tashkent University of Information Technologies named after Muhammad al-Khwarizmi, Tashkent 100084, Uzbekistan; rustammix.rs@gmail.com; 3 Department of Management Theory and Information Security, Samarkand State University named after Sharof Rashidov, Samarkand 140100, Uzbekistan; 4 Department of Artificial Intelligence and Information Systems, Samarkand State University named after Sharof Rashidov, Samarkand 140100, Uzbekistan; isroil.jumanov2019@gmail.com (I.J.); sunatilloxolmonov@gmail.com (S.K.); 5 Department of Computer Engineering, Tashkent University of Applied Sciences, Tashkent 100125, Uzbekistan; toshtemirho@gmail.com; 6 Department of Data Communication Networks and Systems, Tashkent University of Information Technologies, Tashkent 100200, Uzbekistan; dhasanov@tuit.uz (D.K.); kh.khujamatov@tuit.uz (E.H.K.); 7 Department of Digital Pedagogy and Media Education Development, Republican Scientific and Methodological Center for the Development of Education of the Republic of Uzbekistan, Tashkent 100180, Uzbekistan; 8 Department of Applied Informatics and Information Security, Osh State University, Osh 723500, Kyrgyzstan; 9 Department of Information Systems and Technologies, Fergana State Technical University, Fergana 150100, Uzbekistan; 10 Department of Computer and Software Engineering, Jizzakh Polytechnic Institute, Jizzakh 130100, Uzbekistan; 11 Department of Computer Engineering, AI Convergence Research Center, Konkuk University, Chungju 27478, Republic of Korea

**Keywords:** class imbalance, Cretan Pollen Dataset, data augmentation, EfficientNet, Focal Loss, model ensembling, pollen grain classification, reproducibility, Squeeze-and-Excitation, YOLO

## Abstract

Automatic classification of pollen grains in microscopic images supports melissopalynology and honey quality control but is complicated by class imbalance and morphologically similar classes. We evaluated factors affecting classification on the Cretan Pollen Dataset v1 (CPD-1; 20 classes, 4025 images) with YOLOv8s-cls, YOLO11s-cls, YOLO26s-cls, EfficientNet-B3 and a reproduced RCANet. Each configuration was trained with five seeds; hyperparameters and ensembles were selected on validation data only. Augmentation improved the macro F1-score by about 1.2 percentage points; a size-matched unbalanced control showed that this gain came from the larger training set, not from class balancing. The most difficult class, Ceratonia, was misclassified mainly as Vitis and Olea; its F1-score fell from 0.878 to 0.778 when its original training images were reduced from 26 to 10. Focal Loss and Squeeze-and-Excitation blocks did not improve Ceratonia reproducibly (changes in its F1-score from −0.03 to +0.03, not significant after Holm correction), and the YOLO architectures could not be ranked reliably. An ensemble of EfficientNet-B3 and YOLO models selected on validation data outperformed the best single model in all seeds (macro F1-score 0.9720 vs. 0.9631). Several single-run effects were not reproduced with multiple seeds, underlining the need for multi-seed evaluation and validation-only selection.

## 1. Introduction

Pollen analysis is used in a number of applied problems: quality control and determination of the botanical origin of honey (melissopalynology), forecasting of allergen load, forensic examination, palaeoclimatic reconstruction and biodiversity monitoring. The traditional approach to pollen grain identification relies on the visual examination of microscopic preparations by a qualified palynologist; it requires considerable time and highly specialized expertise, which limits its scalability and its routine use, for example, for large-scale honey quality control in the food industry [1,2,3].

The development of deep learning methods, particularly convolutional neural networks (CNNs), has made it possible to automate this task. Early work applied standard classification architectures such as VGG, ResNet and Inception to images of individual pollen grains [4,5,6,7]. In parallel, several studies have addressed micro-object recognition by exploiting redundant information structures and structural characteristics of the images [8,9]. Tsiknakis et al. [10] introduced the Cretan Pollen Dataset (CPD-1), comprising more than 4000 images of 20 pollen types of the Cretan flora collected in a study of monofloral honey, and showed that a soft-voting ensemble of four pretrained CNNs reached an accuracy of 97.5% and a macro F1-score of 96.89% on a hold-out test set. They also reported per-class results and attributed some of the remaining confusions to the shared morphological characteristics of related taxa such as Thymbra, Origanum, Salvia and Satureja. Mahmood et al. [11] proposed an attention-guided feature aggregation network with Squeeze-and-Excitation blocks, and Zolfaghari and Sajedi [12] proposed RCANet, a ResNet18 modified with a Ventral–Dorsal Attention Block inspired by the two-streams hypothesis of the visual cortex, reporting a weighted F1-score of 98.69% on CPD-1 with five-fold cross-validation.

Despite the high aggregate performance reported in these studies, several methodological questions remain open. First, the published results were obtained from single training runs on different, unpublished partitions of CPD-1. Because the aggregate accuracy on this dataset is close to saturation and the most difficult classes contain only a few dozen test images, differences between methods may correspond to a handful of images and may be smaller than the variation between training runs; to our knowledge, this variation has not been quantified for CPD-1. Second, CPD-1 is strongly imbalanced (from 9 to 716 training images per class in our main partition), and offline augmentation is commonly used to balance it [10]. Balancing by augmentation, however, also enlarges the training set and adds transformed images, so its effect has not been separated from that of training-set enlargement [13,14,15]. Third, although per-class results show which classes are difficult [10], it has not been examined experimentally whether the errors in these classes stem mainly from morphological similarity or from the small number of original training specimens. Fourth, methods intended to improve difficult classes, such as loss re-weighting and attention modules, are usually evaluated on a single architecture [16,17], which does not allow a general effect of the method to be distinguished from an architecture-specific one.

The YOLO family of architectures deserves particular attention in this context. Originally developed for real-time object detection, it also provides classification variants (YOLO-cls) that are computationally efficient and available with pretrained weights in several versions (YOLOv8, YOLO11 and YOLO26). This makes them attractive for resource-constrained deployment, for example in portable honey-analysis systems. To our knowledge, their application to pollen classification and the behavior of accuracy-enhancement methods across several YOLO versions have rarely been studied [18,19].

This study addresses these questions with an evaluation protocol in which every configuration is trained with five random seeds and all hyperparameters and ensembles are selected on the validation set only. Specifically, we performed the following:

- Evaluated three YOLO classification models (YOLOv8s-cls, YOLO11s-cls, YOLO26s-cls), EfficientNet-B3 and a reproduction of RCANet on two partitions of CPD-1;
- Separated the effect of class balancing from that of training-set enlargement by means of an augmented but unbalanced control training set;
- Tested Focal Loss and the Squeeze-and-Excitation block, individually and in combination, on all three YOLO architectures;
- Analyzed the errors for the most difficult class, Ceratonia, across models and seeds, and examine the role of the number of original training specimens in a controlled experiment;
- Evaluated ensembles of models from different families, selected on the validation set;
- Assessed the statistical significance of the differences with tests appropriate to the metrics used and with correction for multiple comparisons.

The article is organized as follows: Section 2 reviews related work. Section 3 describes the data (Section 3.1), the augmentation scheme (Section 3.2), the training protocols (Section 3.3) and the statistical analysis (Section 3.4). Section 4 presents the results: baseline experiments (Section 4.1), the effect of training-set augmentation and balancing (Section 4.2), Focal Loss and the SE-Block on YOLOv8s-cls (Section 4.3 and Section 4.4) and across architectures (Section 4.5), comparisons with RCANet, EfficientNet-B3 and published results (Section 4.6), the alternative partition (Section 4.7), ensembles (Section 4.8), the ablation study (Section 4.9) and the analysis of Ceratonia errors (Section 4.10). Section 5 discusses the results, and Section 6 concludes.

A feature of CPD-1 shapes the methodology of this work. Overall accuracy on this dataset is close to saturation: most of the 20 classes are recognized with an F1-score close to 1.0 even by baseline models, and differences between approaches appear mainly in a few small and difficult classes, primarily Ceratonia, as well as Vitis and Pistacia. The main metric of this study is therefore the macro F1-score, which gives equal weight to all classes and is sensitive to the recognition of small classes, whereas the weighted F1-score is dominated by the large classes and tends to mask these differences. Because differences in such classes can amount to only a few test images, all comparisons are based on multiple training runs.

## 2. Related Work

### 2.1. Automatic Classification of Pollen Grains Using Deep Learning Methods

Automatic identification of pollen grains from microscopic images has attracted increasing attention over the past decade. Early work relied on classical machine-learning methods (e.g., support vector machines and random forests) applied to hand-crafted morphometric and texture features such as area, perimeter, eccentricity, Hu moments and texture descriptors [20]. With the transition to CNNs, classification accuracy increased substantially: architectures such as VGG, ResNet and Inception [6,7,21], typically pretrained on ImageNet, were adapted to pollen classification and evaluated on datasets such as POLLEN23E, POLLEN73S and POLLEN13K [4,5,22,23].

Palynological datasets are typically small and imbalanced, reflecting the uneven abundance of pollen types in the samples and the effort required to collect and annotate specimens. Data augmentation [24,25], oversampling of rare classes, and transfer learning from ImageNet-pretrained models [4] are therefore commonly used. As shown in the present study, however, enlarging the training set by augmentation and equalizing the class frequencies are distinct effects that need to be separated.

### 2.2. Cretan Pollen Dataset and Related Work

The Cretan Pollen Dataset v1 (CPD-1) was introduced by Tsiknakis et al. [10] in a study of the botanical origin of the Cretan monofloral honey “Pefkothymaromelo Kritis PDO”. Using a hold-out test set comprising half of the images, they compared four ImageNet-pretrained CNNs and their ensembles; a soft-voting ensemble of all four networks reached an accuracy of 97.5%, a macro F1-score of 96.89% and an AUC of 0.9995. They also reported per-class results and the confusion matrix of this ensemble, and attributed some of the remaining confusions to the shared morphology of related taxa such as Thymbra, Origanum, Salvia and Satureja. Their offline augmentation scheme is adopted in the present study (Section 3.2). In a preliminary test on pollen images obtained from honey sediments, the same model performed poorly, indicating limited transfer from reference images to honey samples.

Mahmood et al. [11] proposed APFA-Net, a lightweight network trained from scratch that combines hierarchical feature aggregation with Squeeze-and-Excitation blocks, and reported an accuracy of 98.33% and an F1-score of 98.39% on CPD-1 with cross-validation. Zolfaghari and Sajedi [12] proposed RCANet, a modification of ResNet18 in which a Ventral–Dorsal Attention Block (VDAB) is integrated into each basic block. The VDAB combines channel attention (the ventral block, addressing “what” is depicted) with spatial attention (the dorsal block, addressing “where” the features are located), following the two-streams hypothesis of the human visual cortex. Using five-fold cross-validation, the authors reported a weighted F1-score of 98.69% on CPD-1, 97.83% on POLLEN13K and 98.24% on POLLEN23E. To our knowledge, independent reproductions of these results have not been reported. 

### 2.3. YOLO Architectures in Image Classification Problems

The YOLO (You Only Look Once) family of architectures, originally developed by Redmon et al. [26] for real-time object detection, includes classification variants (YOLO-cls) in recent versions such as YOLOv8, YOLO11 and YOLO26 [18,27]. These variants use the backbone of the detection models with a classification head instead of a detection head. Although these architectures are widely used for detection, to our knowledge their use for pollen classification has rarely been described. Successive versions differ in their building blocks: YOLOv8 uses C2f blocks, whereas YOLO11 and YOLO26 combine C3k2 blocks with a C2PSA block that applies position-sensitive multi-head self-attention [18,27]. This raises the question of how external attention modules interact with the built-in attention of a given backbone.

### 2.4. Methods for Compensating for Class Imbalance and Difficult Classes

To address class imbalance and hard examples, the Focal Loss proposed by Lin et al. [28] for dense object detection has been widely adopted for classification. It multiplies the cross-entropy by the factor (1 − p_t)^γ, which reduces the contribution of well-classified examples to the gradient and increases the relative weight of difficult ones. The focusing parameter γ is usually tuned empirically for a given task; Lin et al. examined values from 0 to 5 and obtained the best detection results with γ = 2.

A parallel line of work adds attention mechanisms to CNNs to increase the discriminative power of the features. The Squeeze-and-Excitation network (SENet) [29] implements channel attention: the feature map is averaged globally over its spatial dimensions, and a small fully connected network assigns an adaptive weight to each channel. Later work, such as CBAM [30], combined channel attention with spatial attention, which is conceptually close to the VDAB used in RCANet [12]. SE blocks have also been used in a pollen classification network [11].

In the pollen classification literature, these methods have usually been evaluated on a single architecture and with single training runs. It therefore remains unclear whether a reported effect is a general property of the method or specific to a particular architecture or training run. The present study addresses this question by evaluating both methods on three architectures of the same family, with multiple seeds and validation-based hyperparameter selection. 

## 3. Materials and Methods

### 3.1. Data

This study used the publicly available Cretan Pollen Dataset v1 (CPD-1) pollen grain microscopy dataset. CPD-1 contains 4,025 pollen grain images from 20 plant species characteristic of the flora of the island of Crete, Greece. Samples were collected between April 2019 and April 2021 as part of a study on the composition of Cretan monofloral honey “Pefkothymaromelo Kritis PDO”. The dataset is characterized by a pronounced class imbalance (the number of images per class varies from several dozen, e.g., Pinus, Ebenus and Pistacia, to several hundred, e.g., Myrtus and Olea) and by a small proportion of poorly segmented objects [10].

Two partitions of the CPD-1 dataset were used. Dataset 1 is the primary partition, with separate training, validation and test sets, and is used in all experiments except those in Section 4.7. Dataset 2 is an alternative partition of the same 4025 images with a different allocation of images to the training and test sets; it is used in Section 4.7 to assess whether the main findings depend on a particular partition. Because both partitions are drawn from the same image collection, they are not independent datasets. For Dataset 2, a validation subset was drawn once from its training split (15%, stratified by class, with a fixed random seed); this subset is identical for all models. The public release of CPD-1 does not include specimen or slide identifiers, so it cannot be excluded that images originating from the same microscope slide occur in different subsets of either partition. Table 1 shows the distribution of images across the 20 classes for both partitions. For Dataset 1, the column “Train (aug.–unbal.)” additionally gives the size of the augmented–unbalanced control training set used in Section 4.2, in which each class was expanded by a factor of ≈5.12 while preserving the original class proportions; in the balanced training set (Section 3.2), every class contains 716 images. The “Total” column refers to original images only. These data clearly illustrate the pronounced imbalance characteristic of CPD-1.

Class imbalance of this kind is widely regarded as a key challenge in training deep learning models [12,13,14]: a model may become biased towards the dominant classes, reducing the recognition accuracy of rare classes even when aggregate metrics dominated by large classes, such as overall accuracy and the weighted F1-score, remain high. Whether, and to what extent, this applies to CPD-1 is examined in Section 4.1, Section 4.2 and Section 4.10.

### 3.2. Data Augmentation

Given the small number of training images for several classes of CPD-1, in particular Ebenus (10), Pinus (9), Asphodelus (10) and Pistacia (10), offline data augmentation was used to enlarge the training set. We adopted the augmentation scheme of Tsiknakis et al. [10], who introduced CPD-1: the eight transformations, their parameter ranges and their application probabilities listed in Table 2 are identical to those reported in their work. 

The transformations are applied in the order listed in Table 2, and for each generated image, the application of each transformation is decided independently with the given probability. The photometric transformations (Gaussian blur, linear contrast correction and brightness multiplication) are applied with a probability of 30% each; rotation in the range [−180°, 180°] and translation along both axes in the range [−0.2, 0.2] of the image size are always applied, since pollen grains have no fixed orientation in the slide; vertical and horizontal flips are applied with a probability of 50% each. A generated image can therefore be, for example, rotated, translated and horizontally flipped, but not blurred.

Rotation and translation expose image regions that lie outside the original frame. Instead of filling these regions with black, which would introduce an artificial dark border absent from the original micrographs, they are filled with the background color of the image, estimated as the median color of its outermost border pixels. This is the only modification of our implementation relative to the scheme of [10].

Augmentation was performed offline, once, with a fixed random seed (42): all original training images of a class were copied unchanged, and the missing images were generated by cycling through the original images of that class and applying the stochastic transformation pipeline to each. To construct the balanced training set, every class was expanded to the size of the largest class (716 images; 14,320 in total); the same procedure was used to construct the augmented–unbalanced control set of Section 4.2 (Table 1). The validation and test sets were never augmented. It should be noted that augmented copies of a single image are not independent biological samples: for example, the 716 balanced training images of Ceratonia are derived from only 26 original images.

In addition to this offline augmentation, the training pipeline of each model applies its own online augmentations during training. For the YOLO models, these are the default transformations of the Ultralytics classification pipeline (version 8.4.150): RandAugment, random erasing (*p* = 0.4), horizontal flip (*p* = 0.5), HSV jitter (hue 0.015, saturation 0.7, value 0.4), translation (0.1) and scaling (0.5); mosaic and mixup are not used in the classification pipeline. The online augmentations of EfficientNet-B3 and RCANet are described in Section 3.3.

**Figure 1:**
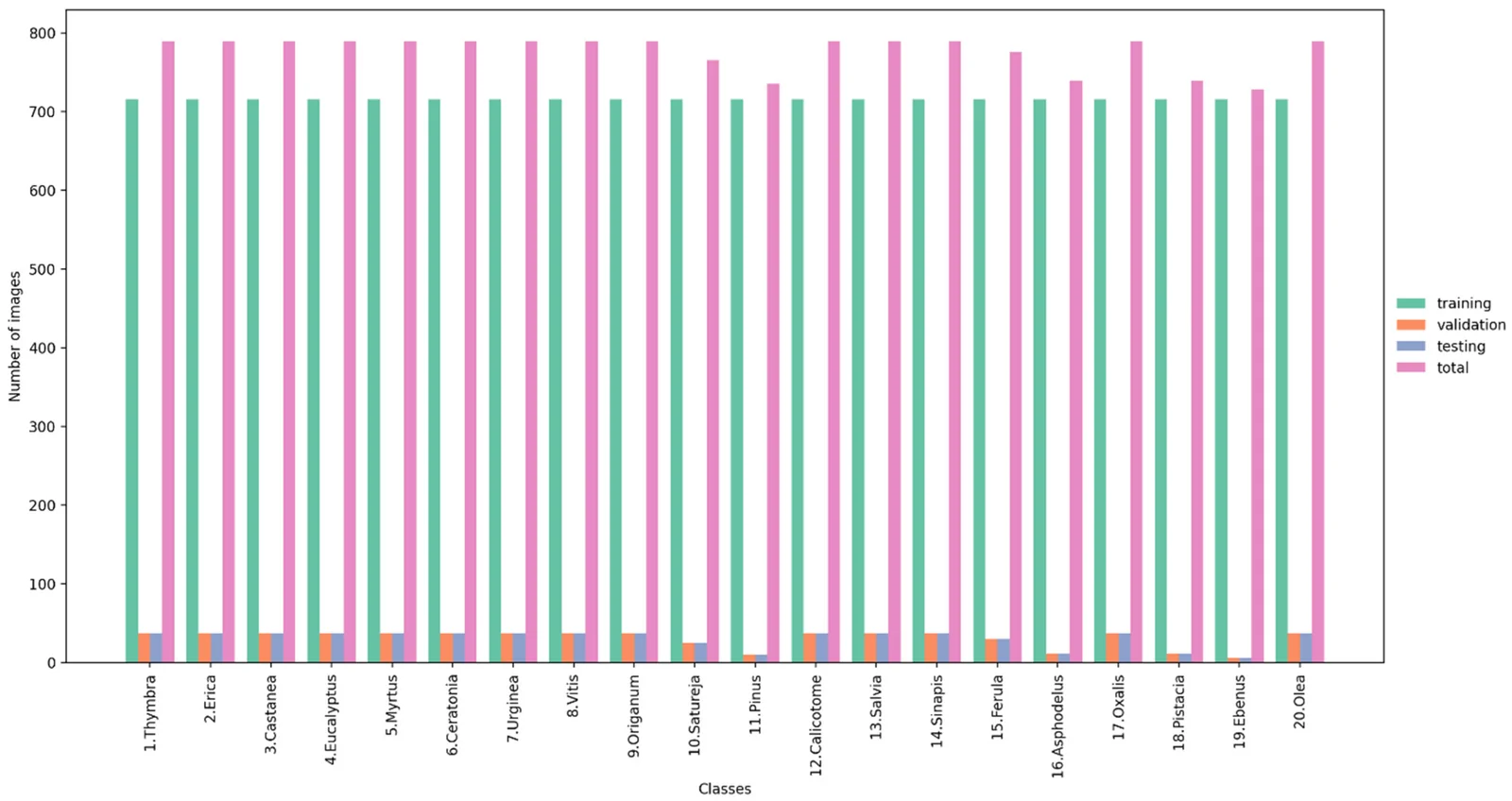
Distribution of images across 20 classes of the CPD-1 dataset after data augmentation.

Figure 1 shows the distribution of images across the 20 classes of CPD-1 after balancing (training, validation and test sets and their sum).

### 3.3. Training Protocol and Implementation Details

All models were trained and evaluated under a unified protocol within each model family; the settings are summarized in Table 3. Each configuration was trained with five random seeds (42, 7, 123, 2024 and 777), and all results are reported as the mean ± standard deviation over seeds. In every experiment, the validation set was used for checkpoint selection and early stopping, and the test set was used only for the final evaluation; no hyperparameter or model selection was performed on the test set.

*YOLO models.* YOLOv8s-cls, YOLO11s-cls and YOLO26s-cls were trained with the Ultralytics library (version 8.4.150), starting from the ImageNet-pretrained classification weights of the corresponding models (the classification layer was replaced by a 20-class layer). All three architectures used the same settings: SGD with an initial learning rate of 0.01, linearly decayed to 1% of its initial value, momentum 0.937, weight decay 5 × 10^−4^ and a 3-epoch warm-up; a batch size of 64; an input size of 224 × 224 pixels; mixed-precision training; and at most 100 epochs with early stopping (patience = 20 epochs). The checkpoint with the highest validation top-1 accuracy was used for evaluation. In the original submission, the Ultralytics default setting optimizer = auto had been used, which selects the optimizer according to the total number of training iterations: AdamW for short schedules and MuSGD for long ones. As a result, the experiments on the unbalanced training set (about 4400 iterations) and those on the balanced training set (about 22,400 iterations) had been run with different optimizers; the training logs of the original balanced experiments confirm that MuSGD was used. In addition, the early-stopping patience had not been the same for all configurations. In the present version, the optimizer and all other settings were fixed explicitly and are identical for all YOLO configurations.

Focal Loss. For a sample with true class t and predicted softmax probability *p_t_*, the Focal Loss [28] is $\mathrm{FL}(p_{t})=-\alpha_{t}{\left(1-p_{t}\right)}^{\gamma }\log(p_{t})$, where γ ≥ 0 is the focusing parameter and *α_t_* is a class weight; the loss is averaged over the mini-batch. In all reported experiments, *α_t_* = 1 for all classes, i.e., no class weighting was applied. The implementation replaces the cross-entropy loss of the Ultralytics classification model and is scaled to the same magnitude as the framework’s cross-entropy loss, so that γ = 0 reproduces it exactly. For γ < 1, the term (1 − *p_t_*) was clipped from below at 10^−6^ for numerical stability, since the gradient of ${\left(1-p_{t}\right)}^{\gamma }$ diverges as *p_t_* → 1. The value of γ was selected separately for each architecture from {0.5, 1.0, 2.0} as the value with the highest mean validation macro F1-score over the five seeds; the selection was made before the test results were inspected.

SE-Block. A Squeeze-and-Excitation block [29] (global average pooling, a fully connected layer reducing the number of channels by a factor of 16 (at least 4), ReLU, a fully connected layer restoring the number of channels, and a sigmoid gate) was inserted between the backbone and the classification head of each YOLO model, after the pretrained weights had been loaded; its parameters were randomly initialized. The same insertion was used in the combined Focal Loss + SE-Block configuration.

EfficientNet-B3. The ImageNet-pretrained EfficientNet-B3 [31] from torchvision was used with a classification head consisting of dropout (0.4), a fully connected layer (1536 → 512), ReLU, batch normalization, dropout (0.2) and a final fully connected layer (512 → 20). It was trained at its native input resolution of 300 × 300 pixels with AdamW (learning rate 10^−4^, weight decay 5 × 10^−4^), a ReduceLROnPlateau schedule on the validation loss (factor 0.5, patience 5), a batch size of 32, mixed precision and at most 100 epochs, with early stopping (patience = 15) and checkpoint selection based on the validation weighted F1-score. Online augmentation consisted of horizontal and vertical flips, rotation (±180°), translation (±10%) and brightness/contrast jitter (±20%), followed by ImageNet normalization. The loss was the standard cross-entropy; in the original submission, a class-weighted cross-entropy had been used on the unbalanced training set, which has been replaced by the standard cross-entropy to match the YOLO experiments.

RCANet. RCANet [12] was implemented as an ImageNet-pretrained ResNet18 with a Ventral–Dorsal Attention Block (reduction = 16, kernel size = 3) inserted after the second batch-normalization layer of every basic block. Following the training settings described for this architecture, it was trained with SGD (learning rate 10^−3^, momentum 0.9, weight decay 5 × 10^−4^), cosine annealing over 100 epochs, a batch size of 32 and no early stopping; the checkpoint with the highest validation accuracy was used. Online augmentation consisted of horizontal and vertical flips, random affine transformations (rotation ± 30°, translation ± 10%, scaling 0.9–1.1, shear ± 10°) and brightness jitter (±30%). Two variants were evaluated: (i) 84 × 84 pixels without input normalization, as in our original reproduction and in the resolution reported for CPD in [12]; and (ii) 224 × 224 pixels with ImageNet normalization, matching the input conditions of the other models.

Reproducibility. For the PyTorch-based models (EfficientNet-B3, RCANet), the random number generators of Python 3.11, NumPy 2.x and PyTorch 2.11.0, the data-loader workers and the cuDNN backend were seeded and set to deterministic mode; for the YOLO models, the Ultralytics deterministic mode was used. One operation in RCANet (the backward pass of adaptive max pooling) has no deterministic CUDA implementation, so RCANet runs with the same seed are not bitwise identical. All experiments were performed on a single NVIDIA GeForce RTX 5060 Ti GPU (16 GB) with Python 3.11, PyTorch 2.11.0, CUDA 12.8 and scikit-learn 1.8.0.

### 3.4. Statistical Analysis

The unit of replication is the training run: each configuration was trained with five independent random seeds and evaluated on the same fixed test set, and all metrics are reported as the mean ± standard deviation over the five runs.

Comparisons of F1-scores. Because the macro F1-score is the primary metric of this study, differences in macro F1-score and in class-level F1-scores (in particular for Ceratonia) were tested directly on the per-run metric values rather than on per-image correctness. Configurations trained independently were compared with two-sided Welch’s *t*-tests (n = 5 runs per configuration, or n = 15 when results were pooled over the three YOLO architectures). When two quantities were derived from the same runs, as in the comparison of an ensemble with its constituent single model for each seed, a two-sided paired *t*-test over seeds was used. For the main effects, the 95% confidence interval of the difference in means is reported in addition to the *p*-value, together with the minimal difference detectable with 80% power at α = 0.05. Unlike the original submission, which reported separate bootstrap confidence intervals [32] for each model, differences between configurations were tested directly.

Comparisons of per-image correctness. To compare two models on the same test images within a seed, the exact McNemar test [33] (two-sided binomial test on the discordant pairs) was used, and the numbers of discordant images, b (correct only for the first model) and c (correct only for the second model), are reported. This test concerns classification accuracy only and was not used to compare F1-scores.

Multiple comparisons. Within each family of related comparisons (for example, Focal Loss and SE-Block versus cross-entropy across the three architectures and two metrics), *p*-values were adjusted with the Holm procedure to control the family-wise error rate at 0.05; the number of comparisons in each family is stated with the results, and both unadjusted and Holm-adjusted *p*-values are reported where relevant.

Small classes. For classes with few test images, exact confusion counts are reported together with 95% Wilson confidence intervals [34] for the class recall, computed with the number of distinct test images as the sample size, since evaluations of the same images in different seeds are not independent.

Model selection. Hyperparameters (the Focal Loss parameter γ) and ensemble configurations were selected exclusively on the validation set; the selected configuration was then evaluated once on the test set.

A non-significant difference was not interpreted as evidence of equivalence; such results are reported together with the confidence interval of the difference. All tests were performed in Python with SciPy at a significance level of 0.05.

## 4. Results

### 4.1. Baseline Experiments on Pollen Grain Classification

The YOLOv8s-cls classification model was chosen as the baseline model for assessing the applicability of YOLO family architectures to the pollen classification problem on the CPD-1 dataset. Unless stated otherwise, all experiments in this paper were conducted on Dataset 1, the primary CPD-1 partition, which includes separate training, validation and test sets; Dataset 2 is used in Section 4.7. Unlike the subsequent experiments using augmentation, the baseline model was trained on the original, unaugmented (unbalanced) training set of Dataset 1 (2799 images), allowing us to assess the effect of class imbalance before applying compensation methods.

The model was trained according to the protocol described in Section 3.3 with five random seeds (42, 7, 123, 2024 and 777); the validation set was used for checkpoint selection and early stopping, and the test set of Dataset 1 (613 unaugmented images) was used only for the final evaluation. All results are reported as the mean ± standard deviation over the five seeds.

Table 4 presents the overall (aggregated) results of the YOLOv8s-cls baseline model, including top-1 and top-5 accuracy, as well as the macro- and weighted-averaged precision, recall and F1-score across all classes.

Table 5 presents detailed classification results for the YOLOv8s-cls baseline model for each of the 20 pollen classes, including precision, recall, F1-score, and the number of test samples (support) for each class. This detail allows us to identify classes for which the baseline model demonstrates the least robustness and assess the impact of training set imbalance on the final classification performance of individual pollen grains.

As shown in Table 5, the lowest recall is observed for Ceratonia (0.7351 ± 0.0673): over the five seeds, 49 of its 185 test predictions were false negatives, whereas only three images of other classes were predicted as Ceratonia (precision = 0.9795). The lowest precision is observed for Vitis (0.8350 ± 0.0337), followed by Thymbra, Asphodelus and Olea (0.867–0.873), indicating that images of other classes are most often absorbed by these classes; as shown in Section 4.10, a large share of the misclassified Ceratonia images is predicted as Vitis or Olea. Ceratonia therefore has the lowest F1-score (0.8381 ± 0.0401), followed by Vitis (0.8980 ± 0.0136). Its seed-to-seed standard deviation is also among the largest of all classes, an order of magnitude larger than that of the aggregate metrics in Table 4.

Castanea, Ebenus and Pinus were classified without errors in all five seeds. For Ebenus and Pinus, however, this result rests on only 6 and 10 test images, respectively: the 95% Wilson confidence interval for a recall of 10/10 is [0.72, 1.00], and for 6/6 it is [0.61, 1.00]. Perfect scores for such small classes are therefore not interpreted as evidence of robustness or of morphological distinctiveness. Exact confusion counts for all classes are provided in Table 6.

The obtained baseline results (macro F1 = 0.9469 ± 0.0062, weighted F1 = 0.9430 ± 0.0059) serve as a starting point for assessing the effect of data augmentation and for the subsequent comparison with the YOLO11s-cls and YOLO26s-cls architectures.

**Figure 2:**
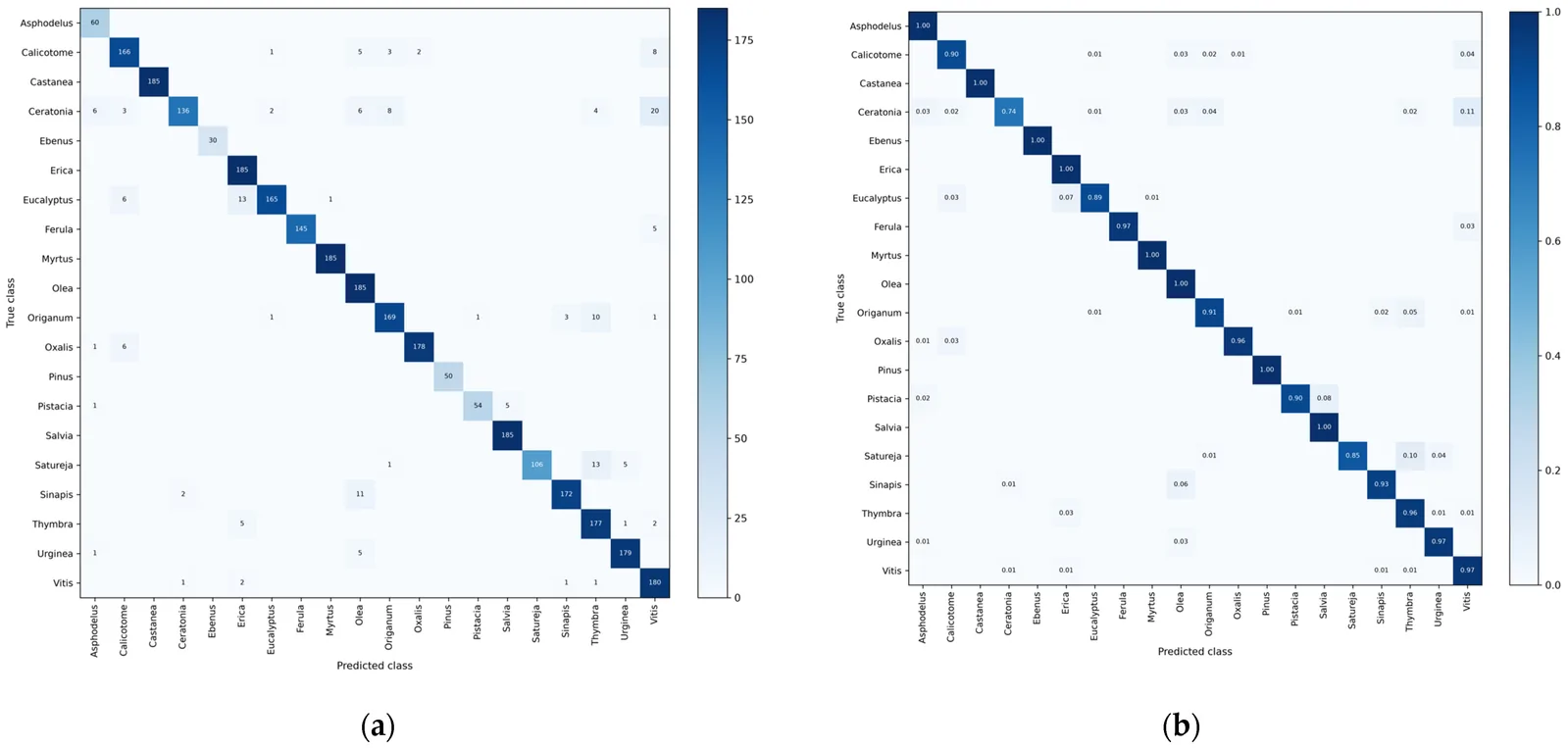
Confusion matrices of the baseline YOLOv8s-cls model on the test set of Dataset 1 (unbalanced training), summed over five seeds: (**a**) absolute counts; (**b**) row-normalized matrix, whose diagonal equals the class recall. Rows correspond to the true class and columns to the predicted class.

Figure 2 shows the confusion matrix of the YOLOv8s-cls baseline on the test set of Dataset 1, summed over the five seeds (5 × 613 = 3065 predictions): (a) absolute counts and (b) the row-normalized matrix, in which each row is divided by the number of test predictions for the true class, so that the diagonal equals the class recall.

As can be seen from the absolute matrix (Figure 2a), most classes are recognized with few errors. Castanea, Ebenus and Pinus were classified correctly in all 185, 30 and 50 predictions, respectively, and Asphodelus, Erica, Myrtus, Olea and Salvia had no false negatives. For Ebenus and Pinus, however, these results rest on only 6 and 10 test images and are therefore not interpreted as evidence of morphological distinctiveness (Table 6). The largest number of errors is observed for Ceratonia (49 false negatives out of 185 predictions), followed by Eucalyptus (20), Calicotome (19), Satureja (19) and Origanum (16). Misclassified Ceratonia images were predicted mainly as Vitis (20 of 49), followed by Origanum (8), Olea (6) and Asphodelus (6), and never as Sinapis or Salvia; conversely, only three images of other classes were predicted as Ceratonia. Other recurrent confusions include Eucalyptus → Erica (13), Satureja → Thymbra (13), Sinapis → Olea (11) and Origanum → Thymbra (10); notably, Satureja, Origanum and Thymbra all belong to the family Lamiaceae. Overall, Vitis (36 false positives), Thymbra (28), Olea (27) and Erica (20) are the classes that most often absorb images of other classes.

The normalized matrix (Figure 2b) shows that the lowest recall is observed for Ceratonia (0.735), followed by Satureja (0.848), Eucalyptus (0.892), Calicotome (0.897) and Pistacia (0.900). The errors are concentrated in a small number of class pairs rather than distributed uniformly, and they recur across seeds. This pattern is consistent with, but does not by itself establish, a morphological origin of the errors; Section 4.10 examines the role of the number of original training images and the characteristics of the consistently misclassified images.

**Figure 3:**
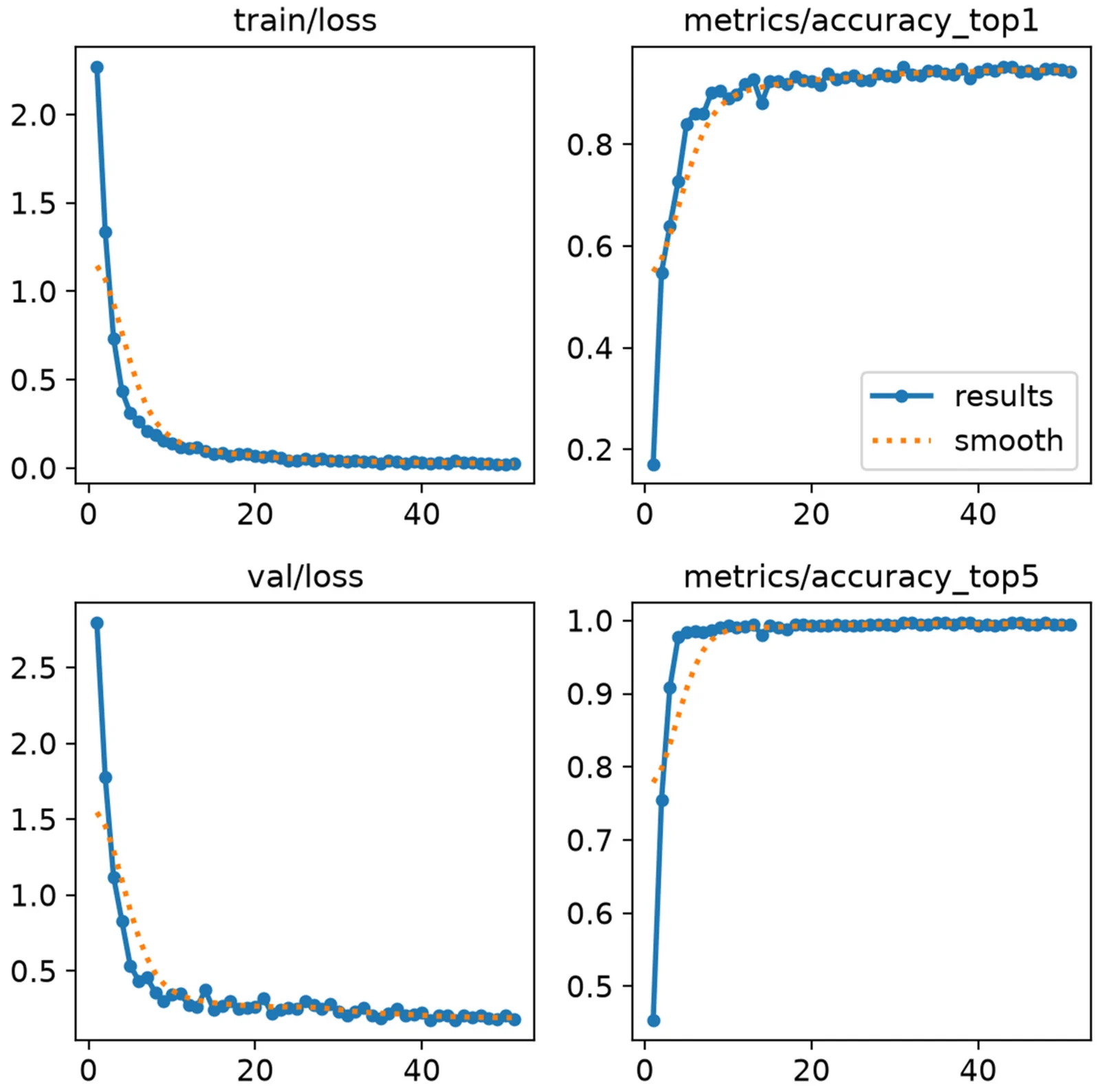
Learning curves of the YOLOv8s-cls baseline (unbalanced training set of Dataset 1, seed 42): loss on the training and validation sets (train/loss, val/loss) and top-1 and top-5 accuracy on the validation set. The best checkpoint was obtained at epoch 31; training was stopped by early stopping after 51 epochs.

Figure 3 shows the learning curves of the YOLOv8s-cls baseline for one representative seed (seed 42): the loss on the training and validation sets and the top-1 and top-5 accuracy on the validation set. The training loss decreases rapidly from 2.27 in the first epoch to below 0.2 by epoch 8 and to approximately 0.03 at the end of training. The validation loss falls from 2.79 to approximately 0.25 by epochs 12–15 and then fluctuates between 0.17 and 0.32 without an upward trend. Validation top-1 accuracy exceeds 0.90 by epoch 8 and remains between 0.92 and 0.95 after epoch 15, while top-5 accuracy is approximately 0.99 or higher from epoch 9 onward. The best validation top-1 accuracy (0.9527) was reached at epoch 31, and training was stopped by early stopping (patience = 20 epochs) after 51 epochs. Across the five seeds, training lasted 51–85 epochs, and the best checkpoint was reached between epochs 31 and 65, indicating that the 100-epoch budget was sufficient for convergence in all runs.

In addition to YOLOv8s-cls, two further baseline models, YOLO11s-cls and YOLO26s-cls, were trained and evaluated under identical conditions, i.e., on the original, unaugmented training set of 2799 images with the protocol of Section 3.3 and the same five seeds. YOLO26s-cls, released by Ultralytics in January 2026, was included as the most recent architecture of the YOLO family at the time of the study. Table 7 compares the results of the three baseline models on the unbalanced data.

Table 8 presents the per-class results of the three baseline models.

**Table 8:** Per-class results of the three baseline models on the unbalanced training set (precision/recall/F1, mean over five seeds). Standard deviations are given in Table 5 (YOLOv8s-cls) and Table 9 (YOLO11s-cls, YOLO26s-cls).

Class	YOLO Models			Support
	YOLOv8s-cls	YOLO11s-cls	YOLO26s-cls	
	(P/R/F1)	(P/R/F1)	(P/R/F1)	
Asphodelus	0.872/1.000/0.931	0.899/1.000/0.946	0.892/0.950/0.919	12
Calicotome	0.918/0.897/0.907	0.929/0.914/0.921	0.933/0.897/0.914	37
Castanea	1.000/1.000/1.000	0.990/1.000/0.995	0.995/1.000/0.997	37
Ceratonia	0.980/0.735/0.838	0.922/0.800/0.855	0.968/0.800/0.876	37
Ebenus	1.000/1.000/1.000	1.000/1.000/1.000	0.971/1.000/0.985	6
Erica	0.903/1.000/0.949	0.935/0.989/0.961	0.964/1.000/0.982	37
Eucalyptus	0.977/0.892/0.932	0.988/0.914/0.949	0.989/0.914/0.949	37
Ferula	1.000/0.967/0.983	1.000/0.960/0.980	1.000/0.967/0.983	30
Myrtus	0.995/1.000/0.997	0.975/1.000/0.987	0.955/1.000/0.977	37
Olea	0.873/1.000/0.932	0.908/1.000/0.951	0.867/1.000/0.928	37
Origanum	0.937/0.914/0.923	0.945/0.935/0.940	0.953/0.881/0.915	37
Oxalis	0.989/0.962/0.975	1.000/0.968/0.984	0.974/0.962/0.968	37
Pinus	1.000/1.000/1.000	1.000/1.000/1.000	1.000/1.000/1.000	10
Pistacia	0.983/0.900/0.939	1.000/0.817/0.899	0.919/0.883/0.897	12
Salvia	0.974/1.000/0.987	0.995/1.000/0.997	0.959/1.000/0.979	37
Satureja	1.000/0.848/0.917	0.974/0.872/0.920	0.934/0.872/0.901	25
Sinapis	0.978/0.930/0.953	0.910/0.951/0.929	0.951/0.935/0.943	37
Thymbra	0.867/0.957/0.909	0.937/0.941/0.938	0.896/0.935/0.913	37
Urginea	0.968/0.968/0.968	0.959/0.995/0.976	0.965/0.968/0.966	37
Vitis	0.835/0.973/0.898	0.877/0.957/0.915	0.873/0.957/0.912	37

**Table 9:** Per-class results of the YOLO11s-cls and YOLO26s-cls baseline models on the unbalanced training set of Dataset 1 (test set, mean ± SD over five seeds). The corresponding values for YOLOv8s-cls are given in Table 5.

Class	YOLO11s-cls			YOLO26S-cls			Support
	Precision	Recall	F1	Precision	Recall	F1	
Asphodelus	0.8989 ± 0.0633	1.0000 ± 0.0000	0.9458 ± 0.0342	0.8917 ± 0.0372	0.9500 ± 0.0745	0.9185 ± 0.0428	12
Calicotome	0.9286 ± 0.0148	0.9135 ± 0.0401	0.9207 ± 0.0236	0.9329 ± 0.0231	0.8973 ± 0.0520	0.9141 ± 0.0311	37
Castanea	0.9895 ± 0.0144	1.0000 ± 0.0000	0.9947 ± 0.0073	0.9947 ± 0.0118	1.0000 ± 0.0000	0.9973 ± 0.0060	37
Ceratonia	0.9219 ± 0.0536	0.8000 ± 0.0622	0.8550 ± 0.0426	0.9677 ± 0.0323	0.8000 ± 0.0242	0.8758 ± 0.0244	37
Ebenus	1.0000 ± 0.0000	1.0000 ± 0.0000	1.0000 ± 0.0000	0.9714 ± 0.0639	1.0000 ± 0.0000	0.9846 ± 0.0344	6
Erica	0.9346 ± 0.0320	0.9892 ± 0.0148	0.9608 ± 0.0179	0.9640 ± 0.0229	1.0000 ± 0.0000	0.9815 ± 0.0117	37
Eucalyptus	0.9884 ± 0.0159	0.9135 ± 0.0226	0.9494 ± 0.0162	0.9886 ± 0.0157	0.9135 ± 0.0226	0.9494 ± 0.0129	37
Ferula	1.0000 ± 0.0000	0.9600 ± 0.0149	0.9795 ± 0.0078	1.0000 ± 0.0000	0.9667 ± 0.0000	0.9831 ± 0.0000	30
Myrtus	0.9745 ± 0.0306	1.0000 ± 0.0000	0.9869 ± 0.0159	0.9552 ± 0.0440	1.0000 ± 0.0000	0.9767 ± 0.0230	37
Olea	0.9078 ± 0.0327	1.0000 ± 0.0000	0.9514 ± 0.0180	0.8667 ± 0.0506	1.0000 ± 0.0000	0.9280 ± 0.0284	37
Origanum	0.9453 ± 0.0192	0.9351 ± 0.0308	0.9401 ± 0.0232	0.9530 ± 0.0165	0.8811 ± 0.0410	0.9154 ± 0.0281	37
Oxalis	1.0000 ± 0.0000	0.9676 ± 0.0121	0.9835 ± 0.0063	0.9739 ± 0.0444	0.9622 ± 0.0148	0.9678 ± 0.0287	37
Pinus	1.0000 ± 0.0000	1.0000 ± 0.0000	1.0000 ± 0.0000	1.0000 ± 0.0000	1.0000 ± 0.0000	1.0000 ± 0.0000	10
Pistacia	1.0000 ± 0.0000	0.8167 ± 0.0373	0.8987 ± 0.0232	0.9192 ± 0.0545	0.8833 ± 0.0745	0.8974 ± 0.0276	12
Salvia	0.9947 ± 0.0118	1.0000 ± 0.0000	0.9973 ± 0.0060	0.9587 ± 0.0137	1.0000 ± 0.0000	0.9789 ± 0.0071	37
Satureja	0.9739 ± 0.0238	0.8720 ± 0.0179	0.9198 ± 0.0093	0.9341 ± 0.0440	0.8720 ± 0.0438	0.9007 ± 0.0237	25
Sinapis	0.9100 ± 0.0510	0.9514 ± 0.0121	0.9294 ± 0.0229	0.9512 ± 0.0292	0.9351 ± 0.0148	0.9429 ± 0.0175	37
Thymbra	0.9365 ± 0.0421	0.9405 ± 0.0352	0.9381 ± 0.0330	0.8960 ± 0.0568	0.9351 ± 0.0730	0.9125 ± 0.0379	37
Urginea	0.9590 ± 0.0287	0.9946 ± 0.0121	0.9763 ± 0.0171	0.9654 ± 0.0536	0.9676 ± 0.0226	0.9655 ± 0.0244	37
Vitis	0.8767 ± 0.0269	0.9568 ± 0.0308	0.9147 ± 0.0232	0.8725 ± 0.0149	0.9568 ± 0.0308	0.9122 ± 0.0073	37

As shown in Table 7, YOLO11s-cls achieved the highest mean macro F1-score on the unbalanced data (0.9521 ± 0.0076), followed by YOLOv8s-cls (0.9469 ± 0.0062) and YOLO26s-cls (0.9451 ± 0.0065). The differences between the architectures, however, are smaller than or comparable to the seed-to-seed standard deviation and are not statistically significant (pairwise Welch’s *t*-tests, *p* > 0.15), so no reliable ranking of the three architectures can be established on this dataset. In the original submission, in which each configuration was trained once with the Ultralytics default optimizer setting, YOLO26s-cls had appeared to be the best architecture on the unbalanced data; this ranking was not reproduced under the unified protocol, which illustrates how sensitive single-run comparisons are to the training configuration.

The per-class results (Table 8) show the same pattern for all three architectures. Ceratonia has the lowest F1-score in every model (0.838–0.876), owing mainly to its low recall (0.735–0.800), whereas its precision remains high (0.922–0.980). Conversely, Vitis, Olea and Thymbra have the lowest precision (0.835–0.937), i.e., they most often absorb images of other classes. The consistency of these patterns across architectures indicates that the difficulty of Ceratonia is not specific to a particular YOLO backbone; its causes are examined in Section 4.10.

### 4.2. Impact of Training-Set Augmentation and Class Balancing on Classification Quality

To evaluate the effect of the data augmentation scheme (Section 3.2), the three YOLO models were retrained on a balanced training set of 14,320 images (716 per class), obtained by offline augmentation of the original training set of Dataset 1. The validation and test sets were left unchanged (613 images each), so the results are directly comparable with the baseline experiments of Section 4.1. All models were trained with the protocol of Section 3.3 and the same five seeds, starting from the ImageNet-pretrained weights of the corresponding classification models.

Moving from the original (2799 images) to the balanced training set changes three factors simultaneously: the class distribution becomes uniform, the number of training images (and hence the number of gradient updates per epoch, from 44 to 224 at a batch size of 64) increases approximately 5.1-fold, and augmented images are added. A comparison of the unbalanced and balanced training sets alone therefore cannot attribute the change in classification quality to class balancing. To separate these factors, a third, control training set was constructed (augmented–unbalanced): each class was expanded by the same augmentation procedure by a factor of approximately 5.12 while preserving the original class proportions, giving the same total of 14,320 images (the per-class sizes are given in Table 1, column “Train (aug.–unbal.)”). The augmented–unbalanced and balanced sets thus have the same size, the same number of updates per epoch and the same augmentation procedure, and differ only in the class distribution.

On the balanced training set, training was stopped by early stopping after 63–76 epochs for YOLOv8s-cls, 58–75 for YOLO11s-cls and 37–70 for YOLO26s-cls, with the best checkpoint reached between epochs 17 and 56 across all architectures and seeds; the 100-epoch budget was therefore sufficient in all runs. Because each epoch on the balanced set contains approximately five times more gradient updates than on the original set, the total number of updates is substantially larger on the balanced set even though the number of epochs is similar (51–100 epochs on the unbalanced set). This difference is taken into account by the control experiment described above.

Table 10 compares the results of YOLOv8s-cls, YOLO11s-cls and YOLO26s-cls on the unbalanced and balanced training sets.

As can be seen from Table 10, all three architectures improve on all metrics when the balanced training set is used. The gain in macro F1-score is largest for YOLOv8s-cls (0.9469 → 0.9631, +0.0162; Welch’s *t*-test, *p* = 0.002; Holm-adjusted over the three architectures, *p* = 0.006), intermediate for YOLO26s-cls (0.9451 → 0.9575, +0.0124; *p* = 0.035; Holm-adjusted *p* = 0.069) and smallest for YOLO11s-cls (0.9521 → 0.9606, +0.0085; *p* = 0.068; Holm-adjusted *p* = 0.069). On the balanced set, the three architectures reach similar macro F1-scores (0.9575–0.9631), and none of the pairwise differences between them is statistically significant (Welch’s *t*-tests, *p* ≥ 0.23), so, as on the unbalanced set, no architecture can be identified as superior. It should be stressed that these gains reflect the combined effect of class balancing, the approximately five-fold larger training set and the added augmented images; the contributions of these factors are separated below.

Table 11 presents the per-class F1-scores of the three architectures on the balanced training set (test set, mean ± SD over five seeds), allowing us to identify the classes for which each architecture shows the highest and lowest performance.

On the balanced training set (Table 11), the per-class F1-scores of Ceratonia are nearly identical across the three architectures (0.895–0.902), whereas its position among the classes differs: it is the lowest-scoring class for YOLOv8s-cls, but Pistacia (0.879–0.919; 12 test images) and, for YOLO26s-cls, Origanum (0.893) score lower for the other architectures. For YOLOv8s-cls, no image of another class was predicted as Ceratonia (precision = 1.000 in all seeds), so all its errors are false negatives, while Vitis remains the class that most often absorbs images of other classes (precision = 0.840).

To determine whether the improvements in Table 10 are due to class balancing itself or to the enlargement of the training set, Table 12 compares the three training sets described at the beginning of this section (test set, mean ± SD over five seeds). The augmented–unbalanced and balanced sets have the same size (14,320 images), number of updates per epoch and augmentation procedure, and differ only in the class distribution.

Table 12 separates the contributions of training-set expansion and class balancing. Pooled over the three architectures (n = 15 runs per training set; four comparisons, Holm-adjusted), expanding the training set with the augmentation scheme while preserving the original class proportions (augmented–unbalanced vs. unbalanced) improved the macro F1-score by 0.0124 (Welch’s *t*-test, *p* < 0.001; Holm-adjusted *p* = 0.003) and the Ceratonia F1-score by 0.033 (*p* = 0.013; Holm-adjusted *p* = 0.039). Once the training-set size, the number of updates and the augmentation procedure were held constant, balancing the class distribution (balanced vs. augmented–unbalanced) produced no detectable additional change in the macro F1-score (−0.0001, *p* = 0.99; Holm-adjusted *p* = 0.99) and a small, non-significant change in the Ceratonia F1-score (+0.009, *p* = 0.38; Holm-adjusted *p* = 0.76). The pattern was not uniform across architectures: for YOLO26s-cls, the augmented–unbalanced set gave a slightly higher macro F1-score than the balanced set, whereas for YOLO11s-cls the opposite was observed; all such differences were within the seed-to-seed variation.

These results indicate that, on CPD-1, the improvement obtained with the balanced training set arises predominantly from the larger and more varied training set rather than from the equalization of class frequencies. In particular, the number of augmented Ceratonia images had little effect: the Ceratonia F1-score was similar with 133 (augmented–unbalanced) and 716 (balanced) training images derived from the same 26 originals. The absence of a detectable balancing effect does not demonstrate that balancing has no effect: the 95% confidence interval for the difference in macro F1-score is [−0.0064, 0.0063] (Ceratonia F1-score: [−0.012, 0.030]), and with 15 runs per training set the experiment had 80% power to detect a difference of approximately 0.009 in macro F1-score (0.029 for Ceratonia). We also note that the gains attributed to class balancing in the original submission (+3.24 and +3.37 pp in macro F1-score for YOLOv8s-cls and YOLO11s-cls) were additionally inflated by the use of different optimizers for the unbalanced and balanced experiments (Section 3.3); under the unified protocol, the corresponding gains are +1.62 and +0.85 pp. Augmented copies of the same image are not independent biological samples; the role of the number of original specimens is examined directly in Section 4.10.

### 4.3. Impact of Focal Loss on the Classification of Difficult Classes

On the balanced training set, Ceratonia and Vitis remained the two classes with the lowest F1-scores for YOLOv8s-cls (0.8950 ± 0.0261 and 0.9082 ± 0.0109, respectively; Table 8). To test whether these classes can be improved by giving more weight to difficult examples during training, the cross-entropy loss (CE) was replaced with the Focal Loss (Section 3.3), which down-weights well-classified examples through the factor (1 − p_t)^γ. YOLOv8s-cls was retrained on the same balanced training set under the protocol of Section 3.3 with three values of the focusing parameter, γ ∈ {0.5, 1.0, 2.0}, each with five seeds; no class-specific weighting was used (α_t = 1). The value of γ was selected on the validation set (Section 3.3).

Table 13 presents the results of the three Focal Loss configurations together with the CE baseline: the validation macro F1-score, which was used to select γ, and the test-set accuracy, macro F1-score, and F1-scores of Ceratonia and Vitis (mean ± SD over five seeds). The per-class F1-scores of all configurations, together with those of the SE-Block configuration discussed in Section 4.4, are given in Table 14.

On the validation set, none of the Focal Loss configurations exceeded the CE baseline (validation macro F1-score 0.9795–0.9813 vs. 0.9821); among them, γ = 0.5 achieved the highest validation score and was therefore selected for the subsequent experiments with YOLOv8s-cls. On the test set, all three Focal Loss configurations yielded a slightly lower macro F1-score than CE (by 0.0038–0.0064), and none of them improved the classification of Ceratonia: its F1-score changed by −0.0018 (γ = 1.0), −0.0200 (γ = 2.0) and −0.0285 (γ = 0.5). For Vitis, the changes ranged from −0.0166 to +0.0052. After Holm adjustment for the nine comparisons in Table 13, none of the differences from CE were statistically significant (adjusted *p* ≥ 0.26).

In all configurations, the errors for Ceratonia remained almost exclusively false negatives: its recall was 0.77–0.82, while its precision was 0.99–1.00 (Table 14). Re-weighting the loss towards difficult examples thus did not enable the model to recognize the Ceratonia images that it otherwise assigns to other classes. These results do not reproduce the finding of the original submission, in which Focal Loss with γ = 1.0 appeared to improve the Ceratonia F1-score from 0.9118 to 0.9275; that difference corresponded to a single additional correctly classified test image in a single run and lies well within the seed-to-seed variation observed here (standard deviation of the Ceratonia F1-score 0.014–0.026). 

### 4.4. Influence of the Attention Mechanism (SE-Block) on the Classification of Difficult Classes

Since re-weighting the loss function (Section 4.3) did not improve the classification of Ceratonia, we tested whether an architectural modification that re-weights the feature channels could help the model extract features that discriminate this class. A Squeeze-and-Excitation block (SE-Block) was inserted between the backbone and the classification head of YOLOv8s-cls (Section 3.3), and the model was trained on the balanced training set under the same protocol and with the same five seeds. Its per-class F1-scores are given in the last column of Table 14.

The SE-Block did not change the classification quality measurably. Its macro F1-score (0.9600 ± 0.0048) was slightly lower than that of CE (0.9631 ± 0.0032; Welch’s *t*-test, *p* = 0.27), and the F1-scores of Ceratonia (0.8920 ± 0.0184 vs. 0.8950 ± 0.0261; *p* = 0.84) and Vitis (0.9008 ± 0.0236 vs. 0.9082 ± 0.0109; *p* = 0.55) were practically unchanged. As with CE and Focal Loss, all errors for Ceratonia were false negatives: its precision was 1.000 in all seeds, whereas its recall was 0.805.

These results do not reproduce the finding of the original submission, in which the SE-Block appeared to reduce the Ceratonia F1-score from 0.9118 to 0.8788 and which led us to conclude that channel attention is unable to capture the spatial features needed to distinguish this class. With five seeds, the effect of the SE-Block on Ceratonia is close to zero and well within the seed-to-seed variation, so the experiment provides no evidence about the relative merits of channel and spatial attention. Whether an architecture that combines channel and spatial attention performs better on this dataset is examined with RCANet in Section 4.6.1.

### 4.5. Architectural Dependence of the Effects of Focal Loss and SE-Block

To test whether the effects observed for YOLOv8s-cls (Section 4.3 and Section 4.4) generalize to other architectures of the YOLO family, Focal Loss and the SE-Block were also applied to YOLO11s-cls and YOLO26s-cls under the same protocol and with the same five seeds. For each architecture, γ was selected on the validation set from {0.5, 1.0, 2.0} (Section 3.3), which gave γ = 0.5 for YOLOv8s-cls and YOLO26s-cls and γ = 1.0 for YOLO11s-cls. Table 15 summarizes the results.

Table 16 lists the differences from CE in macro F1-score and in the Ceratonia F1-score, together with unadjusted and Holm-adjusted *p*-values for the twelve comparisons (three architectures × two methods × two metrics).

Neither method produced a consistent improvement across architectures. For Focal Loss, the change in the Ceratonia F1-score was negative for YOLOv8s-cls (−0.029), negligible for YOLO11s-cls (+0.001) and positive for YOLO26s-cls (+0.029); for the SE-Block, it was −0.003, +0.009 and +0.030, respectively. The changes in macro F1-score were all smaller than 0.007 in absolute value. After Holm adjustment, none of the twelve differences was statistically significant (adjusted *p* ≥ 0.35). The two largest positive effects on Ceratonia were both observed for YOLO26s-cls (Focal Loss and SE-Block, each ≈ +0.03; unadjusted *p* = 0.072 and 0.085); this may indicate an architecture-specific tendency, but it cannot be distinguished from seed-to-seed variation with the present number of runs.

These results do not support the conclusion of the original submission that Focal Loss provides an architecture-independent improvement for Ceratonia, nor the proposed explanation of the architecture-dependent effect of the SE-Block through its interaction with the C2PSA block. That explanation was based on single runs; moreover, the C2PSA block, which implements position-sensitive (multi-head) attention rather than pyramid squeeze attention, is present in both YOLO11s-cls and YOLO26s-cls, so it could not by itself account for a difference between these two architectures. With multiple seeds, the effects of both methods are small relative to the variability between training runs, and their direction depends on the architecture.

### 4.6. Comparison with Alternative Architectures Outside the YOLO Family

In addition to comparing different versions and modifications of the YOLO family, it is of interest how the results of this study relate to previously published results on CPD-1 and how the YOLO classification models compare with widely used classification architectures outside the YOLO family. For this purpose, the specialized attention architecture RCANet was reproduced (Section 4.6.1), and the general-purpose classifier EfficientNet-B3 was evaluated (Section 4.6.2). Each model family was trained with its own established training recipe (Section 3.3, Table 3), whereas all configurations within a family were trained under identical conditions; comparisons between families should therefore be interpreted as comparisons of complete training pipelines rather than of architectures alone.

#### 4.6.1. Reproducing the RCANet Architecture

To verify the results reported by Zolfaghari and Sajedi [12] for RCANet (ResNet18 with the Ventral–Dorsal Attention Block, VDAB), the architecture was implemented as described in the original publication (VDAB after the second batch-normalization layer of every basic block, reduction = 16, kernel size = 3) and trained with the settings given in Section 3.3 (Table 3). In the original submission, RCANet had been trained once, at 84 × 84 pixels and without input normalization, although it was initialized with ImageNet-pretrained weights, which expect normalized inputs. In the present version, two variants were therefore evaluated with five seeds each: (i) RCANet-84, which reproduces the original setting, and (ii) RCANet-224, which uses the same input resolution as the YOLO models and ImageNet normalization. Both variants were trained on the balanced training set of Dataset 1 and on the training set of Dataset 2 (Section 4.7). Because Zolfaghari and Sajedi reported the weighted F1-score, both macro- and weighted F1-scores are given.

Table 17 compares the two RCANet variants with YOLOv8s-cls (CE) and EfficientNet-B3 (Section 4.6.2) trained on the same data.

On Dataset 1, RCANet-224 achieved a macro F1-score of 0.9621 ± 0.0059 and a weighted F1-score of 0.9594 ± 0.0068, on a par with the YOLO models and EfficientNet-B3; none of the differences in macro or weighted F1-score between RCANet-224 and the other four models were statistically significant (Welch’s *t*-tests, *p* ≥ 0.36). The original setting (RCANet-84) yielded slightly lower scores (macro F1 0.9560 ± 0.0074; *p* = 0.19 vs. RCANet-224). On Dataset 2, the difference between the two variants was large (macro F1 0.9502 ± 0.0078 vs. 0.9165 ± 0.0054; *p* < 0.001), indicating that the missing input normalization and the low resolution substantially penalized the pretrained backbone. RCANet-224 performed on a par with the YOLO models on Dataset 2 (*p* ≥ 0.13) and below EfficientNet-B3 (−0.0156, *p* = 0.015). For Ceratonia, RCANet-224 on Dataset 1 (0.8550 ± 0.0208) scored lower than the three YOLO models (by 0.040–0.047; Holm-adjusted *p* = 0.048) and similarly to EfficientNet-B3 (*p* = 0.55).

The statement in the original submission that RCANet is inferior to the YOLO models was based on a single run under the penalized setting and is withdrawn. At the same time, the weighted F1-score of 98.69% reported in [12] was not reached under our protocol: the best mean weighted F1-score of RCANet was 95.94% on Dataset 1 and 95.12% on Dataset 2. The partition of CPD-1 used in [12] has not been published, and the training details available to us may differ from those of the original implementation; this result should therefore be read as a failure to reproduce the reported value under our protocol rather than as evidence against the original study.

#### 4.6.2. Comparison with the Classic CNN Classifier EfficientNet-B3

In addition to specialized attention architectures, it is of interest to compare the YOLO classification models with a standard, general-purpose classification architecture outside the YOLO family. For this comparison, we chose EfficientNet-B3 [31], a widely used convolutional architecture with a favorable accuracy-to-computational-cost ratio across many image classification tasks. The ImageNet-pretrained EfficientNet-B3 was trained with its own established recipe (Section 3.3, Table 3) on the unbalanced and balanced training sets of Dataset 1 and on the training set of Dataset 2, using the same validation and test sets as the YOLO models and the same five seeds.

Table 18 presents the per-class results of EfficientNet-B3 on the three training sets (precision/recall/F1-score, mean over five seeds; the standard deviations of accuracy and macro F1-score are given in the last rows).

As shown in Table 18, Ceratonia is the class with the lowest F1-score for EfficientNet-B3 on all three training sets (0.815, 0.845 and 0.825 on the unbalanced and balanced training sets of Dataset 1 and on Dataset 2, respectively). As for the YOLO models, this is mainly due to their low recall (0.70–0.78), whereas their precision is high on Dataset 1 (0.99). On Dataset 2, its precision is also lower (0.88), i.e., images of other classes are more often predicted as Ceratonia.

On Dataset 1, the balanced training set increased the macro F1-score of EfficientNet-B3 from 0.9561 ± 0.0064 to 0.9653 ± 0.0079 (+0.0092; Welch’s *t*-test, *p* = 0.079) and the Ceratonia F1-score from 0.8148 ± 0.0415 to 0.8448 ± 0.0301 (+0.030; *p* = 0.23). These changes have the same direction as for the YOLO models, but are not statistically significant; as shown in Section 4.2, the improvement obtained with the balanced training set can be attributed mainly to the enlargement of the training set rather than to the equalization of class frequencies.

These results correct the original submission, in which EfficientNet-B3 trained on the balanced set was reported to reach a macro F1-score of 0.9804 and a Ceratonia F1-score of 0.9429, presented as the best result for this class among all methods. Over five seeds, the Ceratonia F1-score of this configuration ranged from 0.806 to 0.879, so the value of 0.9429, obtained in a single run, is not representative. In the original submission, the difference between this value and that of a second, independently retrained instance (0.8788) was also incorrectly described as lying within the run-to-run variability; with five seeds, the standard deviation of the Ceratonia F1-score is 0.030.

Table 19 compares the results of EfficientNet-B3 with the baseline (CE) configurations of YOLOv8s-cls, YOLO11s-cls and YOLO26s-cls on the unbalanced training set of Dataset 1 (2799 training images).

As shown in Table 19, EfficientNet-B3 achieved the highest mean macro F1-score on the unbalanced data (0.9561 ± 0.0064), followed by YOLO11s-cls (0.9521 ± 0.0076), YOLOv8s-cls (0.9469 ± 0.0062) and YOLO26s-cls (0.9451 ± 0.0065). After Holm adjustment for the six comparisons between EfficientNet-B3 and the three YOLO models (two metrics each), none of the differences was statistically significant (unadjusted *p* = 0.027–0.40; adjusted *p* ≥ 0.17). For Ceratonia, EfficientNet-B3 had the lowest mean F1-score of the four models (0.8148 ± 0.0415, compared with 0.838–0.876 for the YOLO models), although these differences were also not significant after adjustment.

Since most experiments in this study use the balanced training set, the corresponding comparison on balanced data is presented in Table 20, together with the best Focal Loss and SE-Block configurations for context.

As shown in Table 20, all configurations trained on the balanced data reach similar macro F1-scores (0.9567–0.9653). EfficientNet-B3 has the highest mean value, but its differences from the three YOLO baselines are not statistically significant (Holm-adjusted *p* ≥ 0.53). For Ceratonia, the picture is the opposite of that reported in the original submission: the two ImageNet-pretrained convolutional networks outside the YOLO family, EfficientNet-B3 (0.8448 ± 0.0301) and RCANet-224 (0.8550 ± 0.0208), have the lowest mean F1-scores of all configurations, whereas the YOLO configurations range from 0.867 to 0.932. Compared with the YOLO baselines trained with CE, the Ceratonia F1-score of EfficientNet-B3 is lower by 0.050–0.057 (unadjusted *p* = 0.013–0.023; Holm-adjusted *p* = 0.080–0.092 over the six comparisons with the three YOLO models). The difference is thus consistent across the three YOLO architectures, but it does not reach significance after correction for multiple comparisons.

The original conclusion that replacing the YOLO backbone with EfficientNet-B3 substantially improves the recognition of Ceratonia, and the suggestion that the YOLO backbones are not suited to extracting the features needed to distinguish this class, are therefore withdrawn; both were based on a single run of EfficientNet-B3 (Section 4.6.2). Because the model families were trained with different pipelines (input resolution, optimizer and online augmentation; Table 3), the present data do not allow the tendency of the YOLO models to recognize Ceratonia somewhat better to be attributed to the architecture itself. The robustness of these observations to a different partition of the data is examined in Section 4.7.

#### 4.6.3. Summary Comparison with Results Published in the Literature

Table 21 brings together the results published for CPD-1 and the main results of this study, including the reproduction of RCANet (Section 4.6.1) and the ensemble selected on the validation set (Section 4.8). For each published result, the metric reported by the authors is given; for this study, the same metrics are given where available.

For the ensemble of Tsiknakis et al. [10], both macro- and weighted-averaged metrics are available, which allows a like-for-like comparison: the ensemble selected on the validation set in this study reaches a similar accuracy (97.26% vs. 97.52%), a slightly higher macro F1-score (97.20% vs. 96.89%) and a slightly lower weighted F1-score (97.24% vs. 97.53%). The F1-scores reported by Mahmood et al. [11] (98.39%) and by Zolfaghari and Sajedi [12] (weighted F1 98.69%) were not reached by any model in this study, including our reproduction of RCANet (Section 4.6.1).

This comparison is indicative only. The published results were obtained with different evaluation protocols: a single hold-out split with half of the images used for testing in [10] and cross-validation in [11,12]. Moreover, the published values come from single training runs, whereas the results of this study are averages over five runs on fixed, explicitly defined partitions. Differences of one to two percentage points between studies with different data partitions and evaluation protocols should therefore not be interpreted as evidence that one method is superior to another.

### 4.7. Robustness of the Results on an Alternative Dataset Partition (Dataset 2)

All experiments presented above were conducted on Dataset 1. To assess whether the main observations depend on this particular partition, namely the absence of a reliable ranking among the YOLO architectures, the difficulty of Ceratonia and the relative performance of EfficientNet-B3, the baseline (CE) configurations of the three YOLO models, EfficientNet-B3 and RCANet-224 were also trained and evaluated on Dataset 2 (Section 3.1, Table 1). Dataset 2 allocates the same 4025 images differently: its training, validation and test subsets contain 2277, 403 and 1345 images, respectively, and the validation subset, drawn once from the training split, is identical for all models. The models were trained on the original (unbalanced) training subset with the protocols of Section 3.3 and the same five seeds. Table 22 compares the results of all models on Dataset 2.

On Dataset 2, as on Dataset 1, the three YOLO architectures could not be distinguished: their macro F1-scores (0.9416–0.9472) did not differ significantly (pairwise Welch’s *t*-tests, *p* ≥ 0.34). EfficientNet-B3 achieved a higher macro F1-score than each of the three YOLO models (by 0.019–0.024; Holm-adjusted *p* = 0.015–0.027 over the six comparisons with the YOLO models), which is a clearer separation than on Dataset 1, where the corresponding differences were not significant (Section 4.6.2). RCANet-224 performed on a par with the YOLO models (Section 4.6.1). For each of the three YOLO models and EfficientNet-B3, Ceratonia had the lowest mean F1-score of all classes on Dataset 2 (0.735–0.825), below the next most difficult class (Thymbra, Origanum or Eucalyptus; 0.874–0.933) by 0.07–0.15, confirming that the difficulty of this class is not specific to the partition of Dataset 1.

Table 23 compares the Ceratonia F1-score on the two partitions under the same training regime, i.e., with the original (unbalanced) training subset in both cases.

The Ceratonia F1-score was lower on Dataset 2 for all three YOLO architectures, significantly so for YOLOv8s-cls and YOLO26s-cls (Holm-adjusted *p* = 0.011 and 0.007), whereas it was practically unchanged for EfficientNet-B3. The two partitions differ in several respects at once (the Ceratonia test subset contains 37 images in Dataset 1 and 50 in Dataset 2, its training subset contains 26 and 42 original images, and the remaining classes are also allocated differently), so the change cannot be attributed to a single factor. What is consistent across both partitions is that Ceratonia remains one of the most difficult classes for every model. In contrast, the result reported for this comparison in the original submission, according to which YOLO11s-cls was particularly robust for Ceratonia, was based on single runs and on a comparison of balanced with unbalanced training; it is not supported by the present data.

In summary, the experiments on the alternative partition support three observations. First, on neither partition can the three YOLO architectures be ranked reliably; the ranking reported in the original submission (YOLO11s-cls > YOLO26s-cls ≈ YOLOv8s-cls) is not supported. Second, Ceratonia is the most difficult class for all models on both partitions. Third, EfficientNet-B3 achieves the highest mean macro F1-score on both partitions, although the difference from the YOLO models is statistically significant only on Dataset 2. For Ceratonia, by contrast, no model family is consistently better: on Dataset 1, the YOLO models tend to score higher than EfficientNet-B3 (Section 4.6.2), whereas on Dataset 2 EfficientNet-B3 scores at least as high as the YOLO models (Table 22). Single-run comparisons of individual classes between architectures or partitions should therefore be interpreted with caution.

### 4.8. Ensembling of Models

The per-class analyses above show that models from different families make different errors: EfficientNet-B3 recognizes Vitis better than the YOLO models, whereas the YOLO models tend to recognize Ceratonia better (Section 4.6.2 and Section 4.7). This creates a basis for ensembling, i.e., combining the predictions of several models so that their errors partly compensate for each other.

The same five base models as in the original submission were used, trained on the balanced training set of Dataset 1: EfficientNet-B3, YOLOv8s-cls (CE), YOLO11s-cls (Focal Loss, γ = 1.0, the value selected on the validation set for this architecture), YOLO11s-cls (SE-Block) and YOLO26s-cls (CE). All 26 combinations of two to five models were evaluated with three strategies, namely soft voting with equal weights (averaging of softmax probabilities), soft voting with weights proportional to the validation macro F1-score of each member, and hard (majority) voting, giving 78 configurations. Each seed was treated as an independent replicate of the whole procedure, i.e., the ensemble for a given seed was built only from the models trained with that seed.

In the original submission, the best configuration had been selected on the test set and then evaluated on the same set, which biases the estimate optimistically. In the present version, the configuration was selected exclusively on the validation set and evaluated once on the test set. Two selection protocols were used: (A) a single configuration with the highest mean validation macro F1-score over the five seeds (main result), and (B) a separate selection for each seed. For comparison, the best single model was selected in the same way as in protocol A. To quantify the bias of the original procedure, the configuration with the highest test macro F1-score in each seed (test selection) was also determined; this value is reported for reference only.

Table 24 presents the results.

Under protocol A, the selected ensemble combined EfficientNet-B3, YOLOv8s-cls (CE), YOLO11s-cls (Focal Loss) and YOLO26s-cls (CE) by hard voting. It reached a macro F1-score of 0.9720 ± 0.0028, compared with 0.9631 ± 0.0032 for the best single model selected on the validation set (YOLOv8s-cls with CE). The ensemble was better in every one of the five seeds (by 0.0053–0.0126; mean difference +0.0089; paired *t*-test over seeds, *p* = 0.0016). It also exceeded the single model with the highest mean test score, EfficientNet-B3 (0.9653 ± 0.0079), by approximately 0.007. Under protocol B, the configuration selected for each seed differed between seeds, but in every seed it combined EfficientNet-B3 with at least one YOLO model; its mean test macro F1-score was 0.9745 ± 0.0022.

Test selection, as in the original submission, gave a mean macro F1-score of 0.9786 ± 0.0013, which is 0.0066 higher than the result of validation-based selection under protocol A. This difference quantifies the optimistic bias of selecting among 78 configurations on the test set; the value of 0.9844 reported in the original submission, obtained by test selection in a single run, should therefore be regarded as an overestimate.

#### Statistical Assessment and Contributions of the Ensemble Components

Consistent with Section 4.1, Section 4.2, Section 4.3, Section 4.4, Section 4.5, Section 4.6 and Section 4.7, the differences in macro F1-score between the individual base models used in the ensembles were small and not statistically significant (Section 4.5 and Section 4.6.2), whereas the ensemble improved on the best single model consistently across all five seeds. A non-significant difference between single models does not, however, establish that they are equivalent; it indicates that, with five seeds and a test set of this size, differences of this magnitude cannot be resolved, which is why comparisons in this study are made with multiple seeds and at the level of individual difficult classes.

At the level of individual test images, the exact McNemar test comparing the protocol-A ensemble with the best single model gave, for the five seeds, discordant counts of b/c = 9/3, 10/0, 11/3, 8/4 and 7/2 (b: images correct only for the ensemble; c: images correct only for the single model). In every seed, more images were corrected than introduced by the ensemble, but the difference was significant only for one seed after Holm adjustment over the five seeds (seed 7: adjusted *p* = 0.010; other seeds: adjusted *p* ≥ 0.23). Within a single seed, the 613 test images are thus insufficient to detect an improvement of this size reliably, whereas the consistent direction over five independent seeds is significant (paired *t*-test, Section 4.8).

To examine which ensembles benefit most, the 78 configurations were grouped by composition (Table 25). Ensembles that included EfficientNet-B3 together with at least one YOLO model outperformed ensembles composed of YOLO models only in every seed, both on the validation set (+0.0036; paired *t*-test over seeds, *p* = 0.0001) and on the test set (+0.0061; *p* = 0.0009). The mixed ensembles mainly improved Vitis (+0.009; *p* = 0.07), whereas YOLO-only ensembles recognized Ceratonia slightly better (0.916 vs. 0.905; *p* = 0.10), consistent with the class-specific strengths of the two families. The number of models had little effect: for mixed ensembles, the mean test macro F1-score was 0.9739, 0.9722, 0.9738 and 0.9731 for two, three, four and five models, respectively. The choice of voting strategy likewise had a negligible effect.

### 4.9. Ablation Study: Joint Effect of Focal Loss and SE-Block

In the previous sections, Focal Loss and the SE-Block were evaluated separately. To test whether their effects combine when they are applied together, YOLOv8s-cls was trained on the balanced training set of Dataset 1 in a 2 × 2 factorial design: with CE or Focal Loss (γ = 0.5, the value selected on the validation set in Section 4.3), each with and without the SE-Block, under the protocol of Section 3.3 and with five seeds per configuration. A single architecture was used so that changes in performance can be attributed to the added components rather than to the backbone; EfficientNet-B3 is discussed separately in Section 4.6.2 and Section 4.8.

In the original submission, the combined configuration had been trained once, with a different optimizer setting (the Ultralytics default optimizer = auto, which selected MuSGD) and a shorter early-stopping patience (15 epochs) than the configurations it was compared with. In the present version, all four configurations were built and trained identically under the protocol of Section 3.3. Table 26 presents the results.

None of the three modified configurations outperformed CE on any of the three metrics. The combined configuration performed between the two single modifications: its macro F1-score equaled that of the SE-Block alone and was higher than that of Focal Loss alone, and its Ceratonia and Vitis F1-scores were also intermediate. A linear model with main effects of Focal Loss and SE-Block and their interaction, fitted to the 20 runs, showed no significant interaction for any metric (interaction terms +0.0064 for macro F1, +0.018 for Ceratonia and +0.022 for Vitis; *p* = 0.13, 0.36 and 0.16, respectively). The point estimates of the interaction are positive, i.e., if anything, the combined configuration lost less than the sum of the losses of the two single modifications.

These results do not reproduce the conclusion of the original submission that Focal Loss and the SE-Block suppress each other when combined. That conclusion was based on single runs and on a combined configuration that was trained with different settings from the single ones. With identical construction and five seeds, neither component improved the classification of difficult classes in YOLOv8s-cls, alone or in combination, and their combination showed no detectable negative interaction.

### 4.10. Analysis of the Classification Errors for Ceratonia

Ceratonia was the most difficult class in all experiments, so its errors were analyzed in more detail in three ways: the structure of the errors across models and seeds, a visual inspection of the misclassified images, and a controlled experiment on the number of original training images.

#### 4.10.1. Structure of the Errors

Table 27 summarizes the misclassifications of the Ceratonia test images for the five models trained with CE on the balanced training set of Dataset 1, summed over the five seeds (185 predictions per model).

The errors have the same structure for all five models. Of the 189 misclassified Ceratonia predictions, 72% were assigned to Vitis or Olea and a further 13% to Origanum, whereas only 4% were assigned to Sinapis or Salvia; the confusion of Ceratonia with Sinapis and Salvia reported in the original submission was based on a confusion matrix computed on the validation set and is not supported by the test-set results. The errors are almost exclusively false negatives: over all models and seeds, only 11 images of other classes were predicted as Ceratonia. The errors are also concentrated in a small number of images. For YOLOv8s-cls, 26 of the 37 Ceratonia test images were classified correctly in all five seeds, five were misclassified in all five seeds, and the remaining six were misclassified in one to four seeds.

#### 4.10.2. Visual Inspection of Consistently Misclassified Images

**Figure 4:**
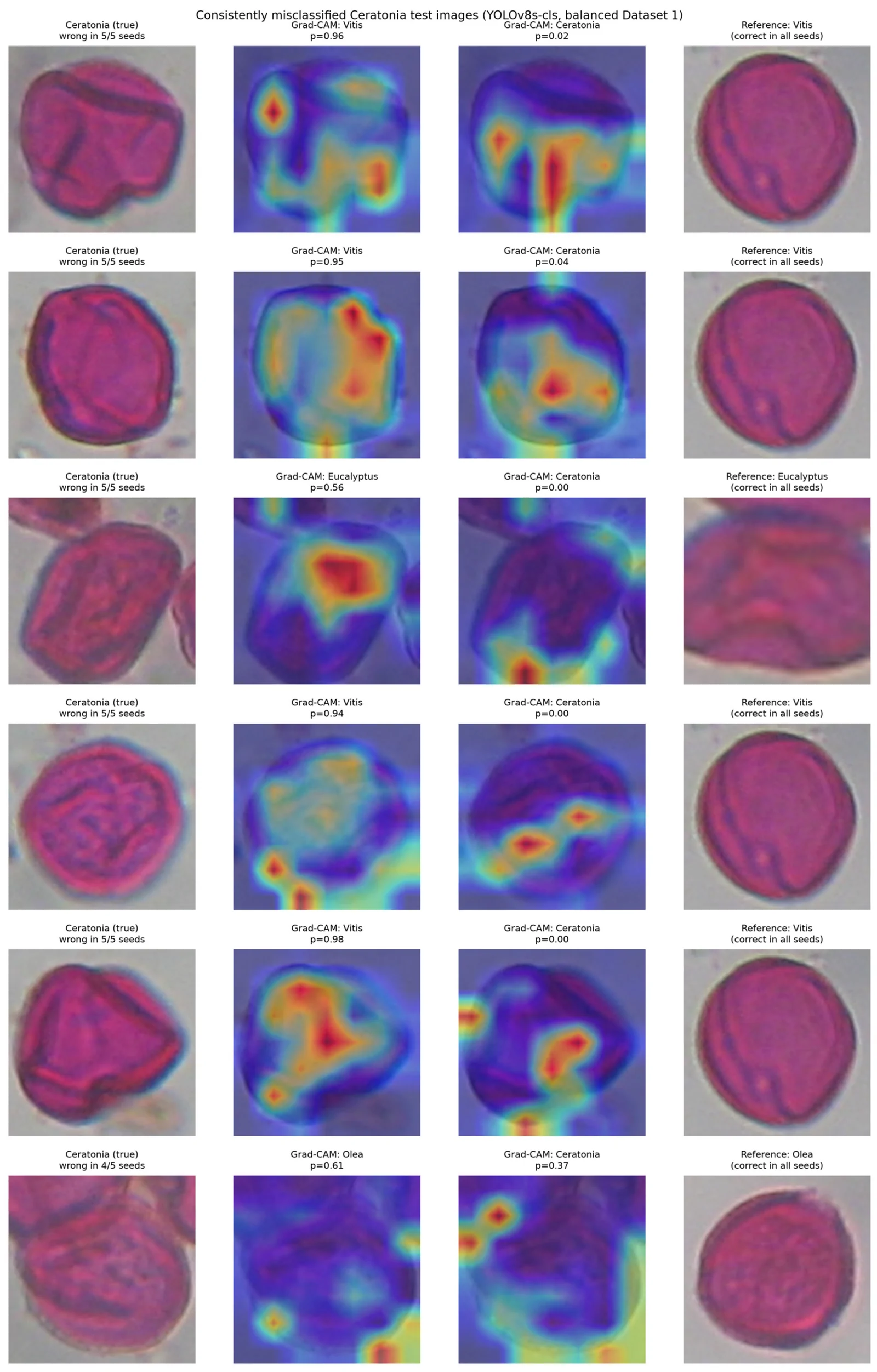
Ceratonia test images misclassified by YOLOv8s-cls (balanced training set, seed 42 for Grad-CAM) in at least four of five seeds. Columns: input image; Grad-CAM for the predicted class; Grad-CAM for Ceratonia; a test image of the predicted class classified correctly in all seeds.

**Figure 5:**
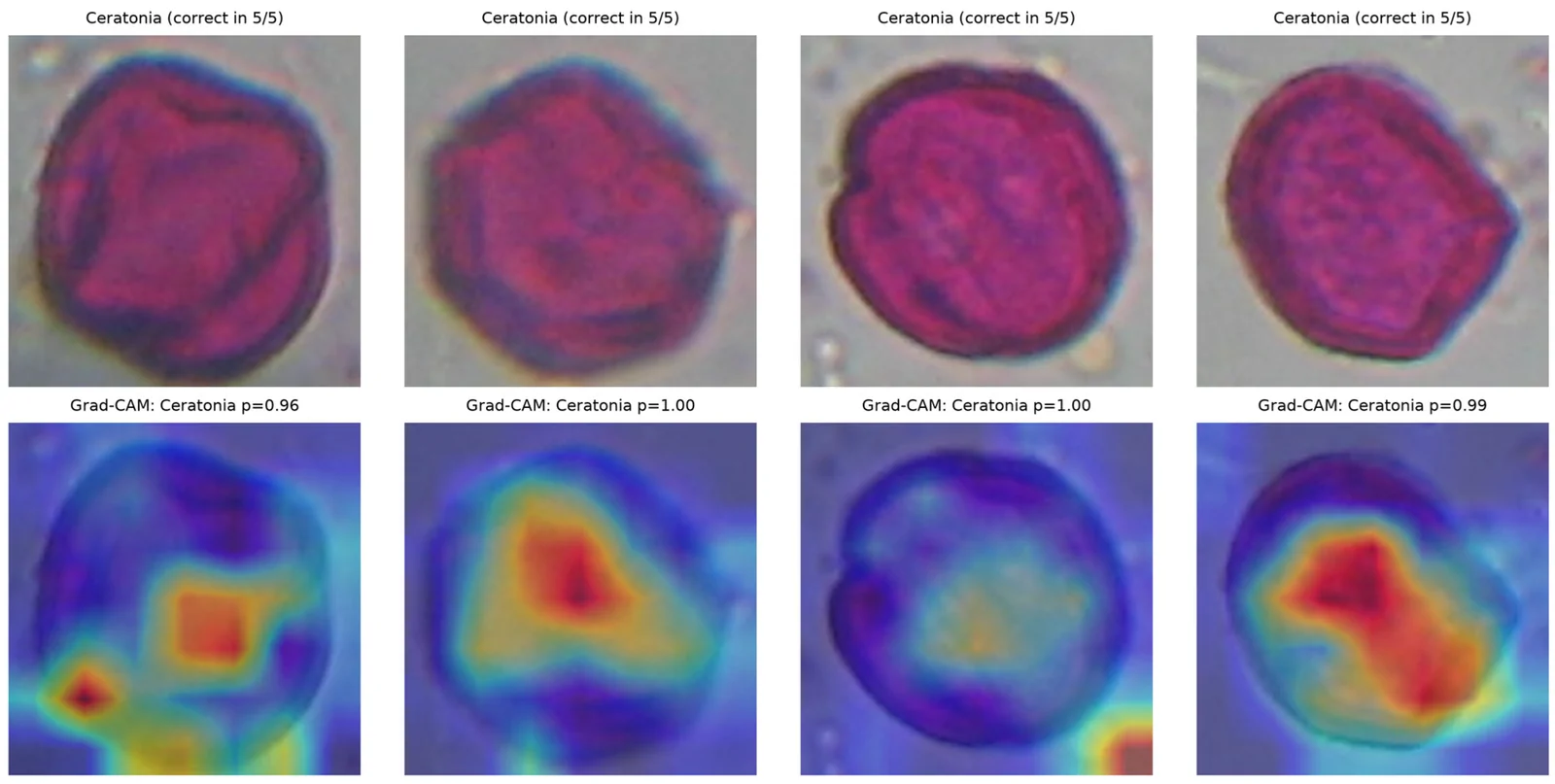
Ceratonia test images classified correctly by YOLOv8s-cls in all five seeds (**top**) and the corresponding Grad-CAM maps for Ceratonia (**bottom**).

Figure 4 shows the six Ceratonia test images misclassified by YOLOv8s-cls in at least four of the five seeds, together with Grad-CAM maps [35] for the predicted and the true class and a correctly classified reference image of the predicted class; Figure 5 shows correctly classified Ceratonia images for comparison.

The misclassified images fall into three groups: (i) deformed or folded grains (two images); (ii) crops containing adjacent or overlapping grains, with partially visible or blurred objects (two images), for which the model was also least confident (maximum probability 0.56–0.61); and (iii) grains without visible artifacts but with a rounded rather than angular outline (two images), which resemble the reference images of Vitis. In contrast, three of the four consistently correct images show the angular, polygonal outline typical of Ceratonia in this dataset. Grad-CAM maps of the misclassified images tended to be more diffuse and occasionally extended to the image border or background; however, similar activations at the border also occurred for some correctly classified images, and the maps, computed on a 7 × 7 feature map, are coarse. They are therefore presented as qualitative illustrations only. Thus, only one of the three groups suggests a genuine visual similarity to the confused class, whereas the other two point to atypical specimens and to segmentation artifacts, which are present in CPD-1 [10]; blurring and distortion of image points are known to impair the identification of micro-objects [36,37].

#### 4.10.3. Effect of the Number of Original Training Images

The analysis above suggests that the training set contains too few examples of the less typical appearances of Ceratonia. To test the role of the number of original specimens directly, three training sets were constructed from the balanced training set of Dataset 1 that differed only in the original Ceratonia images: all 26, a random subset of 18, or a subset of 10 of these (nested subsets), each expanded to 716 images with the augmentation procedure of Section 3.2, while all other classes and the validation and test sets were left unchanged. YOLOv8s-cls was trained on each set with five seeds. Table 28 presents the results.

The Ceratonia F1-score decreased steadily with the number of original images, from 0.878 with 26 images to 0.778 with 10 images (difference 0.100; Welch’s *t*-test, Holm-adjusted *p* = 0.014 over the three pairwise comparisons). A linear regression over the 15 runs gave a slope of +0.0062 per original image (95% CI 0.0030–0.0095; *p* = 0.001), and the curve did not level off at 26 images. The F1-score of Vitis decreased as well (slope +0.0030 per image; *p* = 0.010), consistent with the observation that misclassified Ceratonia images are mostly assigned to Vitis. Notably, all three sets contained the same number of augmented Ceratonia images (716), so the decrease reflects the reduced diversity of the original specimens rather than a reduced number of training images; the same conclusion follows from Section 4.2, where the number of augmented Ceratonia images (133 vs. 716 derived from the same 26 originals) had little effect. The set with all 26 images reproduces the original balanced training set except for the random draws of the augmentation; its Ceratonia F1-score did not differ significantly from that of the original set (0.878 vs. 0.895; *p* = 0.45), although its macro F1-score was slightly lower (0.9567 vs. 0.9631; *p* = 0.041, unadjusted), indicating that the random realization of the augmentation itself contributes some variability.

Taken together, these results indicate that the difficulty of Ceratonia is associated primarily with the small number and limited diversity of its original training specimens, which augmentation cannot compensate for, and with a few atypical or poorly segmented test images. Visual similarity to Vitis may contribute to a subset of images, but our experiments do not isolate its effect, and an assessment of the diagnostic features would require palynological expertise. This interpretation is consistent with the results of Tsiknakis et al. [10], who used a partition with about twice as many original Ceratonia training images and reported a recall of 0.96 for this class, although other differences between the two studies preclude a direct comparison.

## 5. Discussion

This study examined the factors that affect the classification of pollen grains on the CPD-1 dataset, with a focus on difficult, morphologically similar classes. Its main finding is methodological: when each configuration is trained with several seeds and hyperparameters and ensembles are selected on the validation set only, most of the effects reported in the original submission on the basis of single runs, including the improvement of Ceratonia by Focal Loss and the superiority of EfficientNet-B3 for this class, are not reproduced. The effects that remain are an improvement from enlarging the training set with augmentation and from combining models of different families, together with a strong dependence of the difficult class Ceratonia on the number of its original training images.

### 5.1. Training-Set Expansion Rather than Class Balancing

Training on the balanced, augmented training set improved the macro F1-score of all architectures (Section 4.2). The control experiment with an augmented but unbalanced training set of the same size showed, however, that this improvement was due almost entirely to the larger and more varied training set (+1.24 pp in macro F1-score, +3.3 pp for Ceratonia), whereas equalizing the class frequencies had no detectable additional effect (95% confidence interval for the difference in macro F1-score: −0.64 to +0.63 pp). In the original submission, the gain had been attributed to class balancing and had been overestimated (+3.2–3.4 pp for YOLOv8s-cls and YOLO11s-cls), partly because the unbalanced and balanced experiments had been run with different optimizers (Section 3.3). This finding agrees with the observation that class imbalance alone is not necessarily harmful when the minority classes are well separated [12,13,14]: in CPD-1, several of the smallest classes (Pinus, Ebenus) were recognized without errors in most runs even without balancing. Offline augmentation is therefore useful for CPD-1, but its benefit should be attributed to the increased variety of the training images rather than to the equal class frequencies, and it is bounded by the diversity of the original specimens (Section 5.2).

### 5.2. The Difficult Class Ceratonia: Limited Original Specimens and Partial Visual Similarity

Ceratonia was the most difficult class for all models and on both partitions, with errors consisting almost exclusively of false negatives. Contrary to the interpretation in the original submission, these errors are not mainly confusions with Sinapis and Salvia, but assignments to Vitis, Olea and Origanum (Table 27). The controlled experiment in Section 4.10.3 showed that the Ceratonia F1-score depends strongly on the number of original training images (−0.10 when reducing them from 26 to 10, at a constant number of augmented images), and that it had not leveled off at 26 images. Visual inspection showed that the consistently misclassified images are atypical specimens (deformed or folded grains), crops affected by segmentation artifacts, or grains whose rounded outline resembles that of Vitis. These observations suggest that the 26 original images do not cover the variability in appearance of Ceratonia grains, and that augmentation, which only transforms the existing images geometrically and photometrically, cannot create the missing appearances. Morphological similarity to Vitis may contribute to some images, but it is not the only, or demonstrably the main, cause. In the original publication of CPD-1, with about twice as many original Ceratonia training images, a much higher recall was reported for this class [10], which is consistent with this interpretation, although the two studies also differ in other respects.

### 5.3. Loss Re-Weighting and Attention Modules: No Reproducible Effect

Neither Focal Loss nor the SE-Block improved the classification of difficult classes reproducibly. With γ selected on the validation set, the change in the Ceratonia F1-score ranged from −0.029 to +0.030 across the three YOLO architectures; its direction depended on the architecture, and none of the twelve comparisons with CE was significant after Holm adjustment (Section 4.5). The combination of both methods showed neither a cumulative benefit nor a negative interaction (Section 4.9). In all configurations, the errors for Ceratonia remained false negatives, which is consistent with the interpretation in Section 5.2: re-weighting the loss or recalibrating feature channels changes how the model uses the available training examples, but it cannot supply appearances of the class that are absent from the training set. The architecture-dependent effect of the SE-Block reported in the original submission, and its explanation through an interaction with the C2PSA block, were based on single runs and are withdrawn; the C2PSA block, which implements position-sensitive attention, is present in both YOLO11 and YOLO26. These negative results do not exclude small effects: with five seeds, differences in the Ceratonia F1-score smaller than about 0.03 cannot be resolved, and the consistent positive tendency of both methods for YOLO26s-cls (≈+0.03) may merit testing with more runs.

### 5.4. Architectures and Ensembles

Within the YOLO family, the three architectures could not be ranked reliably on either partition, so the rankings reported in the original submission do not hold. EfficientNet-B3 achieved the highest mean macro F1-score on both partitions, but the difference was statistically significant only on Dataset 2, and for Ceratonia no model family was consistently better (Section 4.6.2 and Section 4.7). Because the families were trained with different pipelines, these differences characterize complete training pipelines rather than architectures alone. The reproduced RCANet performed on a par with the other models once the inputs were normalized and the same resolution was used; the original reproduction had been penalized by the missing normalization of the inputs to a pretrained backbone. The weighted F1-score of 98.69% reported for RCANet [12] was not reached, which may reflect differences in the evaluation protocol (cross-validation in [12]) and in implementation details. The most robust improvement was obtained by ensembling. An ensemble selected on the validation set outperformed the best single model in all five seeds (+0.89 pp in macro F1-score), and ensembles combining EfficientNet-B3 with YOLO models outperformed YOLO-only ensembles, consistent with the different class-specific strengths of the two families. Selecting the ensemble on the test set, as in the original submission, overestimated its performance by about 0.7 pp.

### 5.5. Methodological Implications

The revision of this study illustrates how easily single-run comparisons can mislead on a saturated dataset such as CPD-1. The seed-to-seed standard deviation of the Ceratonia F1-score (0.014–0.045) was of the same order as, or larger than, the effects of the methods compared, and a difference of one correctly classified test image changes this score by about 0.016. Several conclusions of the original submission were reversed or not reproduced when five seeds were used. These included the improvement of Ceratonia by Focal Loss, the superiority of EfficientNet-B3 for this class, the negative interaction between Focal Loss and the SE-Block, the inferiority of RCANet, and the size of the effect attributed to class balancing. Two further sources of bias were identified: implicit framework defaults (the automatic optimizer selection, which changed the optimizer between experiments) and the selection of configurations on the test set. For studies on pollen datasets of this size, we therefore recommend training each configuration with several seeds, fixing all training settings explicitly, selecting hyperparameters and ensembles on a validation set only, testing class-level metrics directly with appropriate paired or unpaired tests and corrections for multiple comparisons, and reporting confidence intervals rather than interpreting non-significant differences as equivalence.

### 5.6. Practical Recommendations

For the automated analysis of pollen in honey, the results suggest four practical recommendations. First, offline augmentation is a useful, inexpensive way to enlarge the training set, but its benefit should not be expected from balancing the class frequencies as such. Second, for difficult classes such as Ceratonia, collecting additional original specimens covering their range of appearances is likely to be more effective than modifications of the loss function or the architecture; within the range studied, each additional original image increased the Ceratonia F1-score by about 0.006. Third, combining models from different families gives a modest but consistent improvement and can be recommended when the additional inference cost is acceptable. Fourth, careful segmentation of individual grains is important, since some of the persistent errors arose from crops containing adjacent or overlapping grains.

### 5.7. Limitations of the Study

Several limitations should be noted. First, all experiments were performed on a single dataset; Dataset 1 and Dataset 2 are two partitions of the same images rather than independent datasets, and because the public release of CPD-1 contains no slide or specimen identifiers, images from the same slide may occur in both the training and the test subsets. Second, with five seeds per configuration, the experiments can detect differences of about 0.009 in macro F1-score and about 0.03 in the Ceratonia F1-score with 80% power; smaller effects cannot be excluded. Third, the model families were trained with different, family-specific pipelines, so comparisons between families do not isolate the effect of the architecture. Fourth, Focal Loss and the SE-Block were tested in a single configuration each (one insertion point of the SE-Block, three values of γ, no class weighting), and other variants might behave differently. Fifth, the controlled experiment on the number of original images was performed for one class and one architecture. Finally, the visual analysis of the misclassified images and the Grad-CAM maps are qualitative, and the diagnostic relevance of the observed features was not assessed by a palynologist.

### 5.8. Future Research Directions

Promising directions include the following: (1) collecting additional original specimens of difficult classes and quantifying how many are needed to reach the performance of the other classes; (2) an expert palynological analysis of the misclassified images, including the quality of segmentation, to separate morphological similarity from specimen and imaging artefacts; (3) evaluation on additional datasets (e.g., POLLEN73S [23], POLLEN23E [4]) and with repeated partitions or cross-validation, with specimen-level separation of the training and test data where identifiers are available; and (4) evaluation on pollen images obtained directly from honey sediments, on which models trained on reference images have been reported to perform poorly [10].

## 6. Conclusions

This paper presents a systematic study of the factors affecting the classification of pollen grains on the CPD-1 dataset, with a focus on difficult, morphologically similar classes, using three YOLO classification models (YOLOv8s-cls, YOLO11s-cls and YOLO26s-cls), EfficientNet-B3 and a reproduction of RCANet. All configurations were trained with five random seeds under explicitly fixed protocols, and all hyperparameters and ensembles were selected on the validation set only.

Enlarging the training set with offline augmentation improved the macro F1-score of all architectures. A control experiment with an augmented but unbalanced training set of the same size showed that this improvement (about 1.2 percentage points) was due to the larger and more varied training set, whereas equalizing the class frequencies had no detectable additional effect.

Ceratonia was the most difficult class for all models and on both partitions of the dataset. Its errors were almost exclusively false negatives, assigned mainly to Vitis, Olea and Origanum. A controlled experiment showed that its F1-score depends strongly on the number of original training images: it decreased from 0.878 to 0.778 when the number of original images was reduced from 26 to 10, at a constant number of augmented images. The consistently misclassified images were atypical or poorly segmented grains, or grains whose rounded outline resembles Vitis. The difficulty of this class is therefore associated primarily with the limited number and diversity of its original specimens, with morphological similarity contributing for a subset of images.

Neither loss re-weighting (Focal Loss) nor channel attention (SE-Block), alone or in combination, improved the classification of difficult classes reproducibly: their effects depended on the architecture and were not statistically significant after correction for multiple comparisons. The three YOLO architectures could not be ranked reliably; EfficientNet-B3 achieved the highest mean macro F1-score, and the reproduced RCANet performed on a par with the other models under matched input conditions but did not reach the performance reported in the literature. The most robust improvement came from ensembling: an ensemble selected on the validation set outperformed the best single model in all seeds (macro F1-score 0.9720 vs. 0.9631), and ensembles combining EfficientNet-B3 with YOLO models outperformed YOLO-only ensembles.

A central finding of this work is methodological. Several conclusions drawn from single training runs in the original version of this study were reversed or could not be reproduced with multiple seeds. These included the improvement of Ceratonia by Focal Loss, the superiority of EfficientNet-B3 for this class and the size of the effect of class balancing; selecting the ensemble on the test set overestimated its performance. On saturated pollen datasets of this size, method comparisons should therefore be based on multiple training runs, validation-only model selection, explicitly fixed training settings and class-level statistical testing. For practical applications, collecting additional original specimens of difficult classes and combining models of different families appear more promising than modifications of the loss function or the architecture.

## Figures and Tables

**Table 1:** Distribution of pollen grain images by classes in the two partitions of the CPD-1 dataset.

No.	Class	Dataset 1				Dataset 2				
		Train	Train (Aug.-Unbal.)	Val	Test	Total	Train	Val	Test	Total
1	Thymbra	72	368	37	37	146	62	11	73	146
2	Erica	107	547	37	37	181	94	17	70	181
3	Castanea	144	737	37	37	218	126	22	70	218
4	Eucalyptus	96	491	37	37	170	85	15	70	170
5	Myrtus	712	3643	37	37	786	535	94	157	786
6	Ceratonia	26	133	37	37	100	42	8	50	100
7	Urginea	35	179	37	37	109	50	9	50	109
8	Vitis	195	998	37	37	269	144	25	100	269
9	Origanum	97	496	37	37	171	86	15	70	171
10	Satureja	21	108	25	25	71	31	5	35	71
11	Pinus	9	46	10	10	29	12	2	15	29
12	Calicotome	224	1146	37	37	298	168	30	100	298
13	Salvia	104	532	37	37	178	92	16	70	178
14	Sinapis	123	629	37	37	197	82	15	100	197
15	Ferula	23	118	30	30	83	35	6	42	83
16	Asphodelus	10	51	12	12	34	14	3	7	24
17	Oxalis	65	333	37	37	139	59	10	70	139
18	Pistacia	10	51	12	12	34	14	3	17	34
19	Ebenus	10	51	6	6	22	9	2	11	22
20	Olea	716	3663	37	37	790	537	95	158	790
	Total	2799	14,320	613	613	4025	2277	403	1345	4025

**Table 2:** Augmentation methods used in the study.

Augmentation Method	Hyperparameters	Probability
Gaussian Blur	Sigma [0, 0.3]	30%
Linear Contrast Correction	Alpha [0.75, 1.25]	30%
Brightness Multiplication	Factor [0.7, 1.3]	30%
Rotation	Degrees [−180, 180]	100%
X-Axis Shift	Percentage shift [−0.2, 0.2]	100%
Y-Axis Shift	Percentage shift [−0.2, 0.2]	100%
Vertical Flip	-	50%
Horizontal Flip	-	50%

**Table 3:** Training protocols of the three model families. Within each family, all configurations and all training sets used identical settings.

Setting	YOLO (v8s/11s/26s-cls)	EfficientNet-B3	RCANet
Initialization	ImageNet-pretrained	ImageNet-pretrained	ImageNet-pretrained ResNet18
Input size	224 × 224	300 × 300	84 × 84 or 224 × 224
Input normalization	Ultralytics default	ImageNet	none (84)/ImageNet (224)
Optimizer	SGD, momentum 0.937	AdamW	SGD, momentum 0.9
Learning rate, schedule	0.01, linear decay to 1%, 3-epoch warm-up	10^−4^, ReduceLROnPlateau (0.5, 5)	10^−3^, cosine annealing
Weight decay	5 × 10^−4^	5 × 10^−4^	5 × 10^−4^
Batch size	64	32	32
Max. epochs/early stopping	100/patience 20	100/patience 15	100/none
Checkpoint criterion	val. top-1 accuracy	val. weighted F1	val. accuracy
Loss	CE, Focal Loss	CE	CE
Mixed precision	yes	yes	no
Online augmentation	Ultralytics default (Section 3.2)	flips, rotation, translation, brightness/contrast	flips, affine, brightness
Seeds	42, 7, 123, 2024, 777	same	same

**Table 4:** Overall results of the baseline YOLOv8s-cls model on the test set (unbalanced training set; mean ± SD over five seeds).

Metrics	Macro	Weighted
Top-1 accuracy	0.9436 ± 0.0057	-
Top-5 accuracy	0.9941 ± 0.0019	-
Precision	0.9524 ± 0.0062	0.9484 ± 0.0051
Recall	0.9471 ± 0.0053	0.9436 ± 0.0057
F1-score	0.9469 ± 0.0062	0.9430 ± 0.0059

**Table 5:** Per-class results of the baseline YOLOv8s-cls model on the test set (mean ± SD over five seeds).

Class	Precision	Recall	F1	Support
Asphodelus	0.8721 ± 0.0521	1.0000 ± 0.0000	0.9310 ± 0.0299	12
Calicotome	0.9184 ± 0.0337	0.8973 ± 0.0444	0.9069 ± 0.0250	37
Castanea	1.0000 ± 0.0000	1.0000 ± 0.0000	1.0000 ± 0.0000	37
Ceratonia	0.9795 ± 0.0188	0.7351 ± 0.0673	0.8381 ± 0.0401	37
Ebenus	1.0000 ± 0.0000	1.0000 ± 0.0000	1.0000 ± 0.0000	6
Erica	0.9027 ± 0.0156	1.0000 ± 0.0000	0.9488 ± 0.0086	37
Eucalyptus	0.9766 ± 0.0241	0.8919 ± 0.0191	0.9322 ± 0.0183	37
Ferula	1.0000 ± 0.0000	0.9667 ± 0.0000	0.9831 ± 0.0000	30
Myrtus	0.9947 ± 0.0118	1.0000 ± 0.0000	0.9973 ± 0.0060	37
Olea	0.8731 ± 0.0233	1.0000 ± 0.0000	0.9321 ± 0.0133	37
Origanum	0.9365 ± 0.0389	0.9135 ± 0.0483	0.9232 ± 0.0097	37
Oxalis	0.9892 ± 0.0148	0.9622 ± 0.0148	0.9753 ± 0.0061	37
Pinus	1.0000 ± 0.0000	1.0000 ± 0.0000	1.0000 ± 0.0000	10
Pistacia	0.9833 ± 0.0373	0.9000 ± 0.0373	0.9391 ± 0.0241	12
Salvia	0.9737 ± 0.0000	1.0000 ± 0.0000	0.9867 ± 0.0000	37
Satureja	1.0000 ± 0.0000	0.8480 ± 0.0522	0.9170 ± 0.0315	25
Sinapis	0.9778 ± 0.0232	0.9297 ± 0.0148	0.9530 ± 0.0120	37
Thymbra	0.8674 ± 0.0644	0.9568 ± 0.0148	0.9088 ± 0.0360	37
Urginea	0.9677 ± 0.0114	0.9676 ± 0.0121	0.9676 ± 0.0074	37
Vitis	0.8350 ± 0.0337	0.9730 ± 0.0270	0.8980 ± 0.0136	37

**Table 6:** Exact confusion counts of the baseline YOLOv8s-cls model on the test set of Dataset 1 (unbalanced training), summed over five seeds, and 95% Wilson confidence intervals for the class recall.

Class	Support (n)	TP (Σ5)	FP (Σ5)	FN (Σ5)	Mean Recall	95% CI (Recall)
Asphodelus	12	60	9	0	1.000	[0.76, 1.00]
Calicotome	37	166	15	19	0.897	[0.76, 0.96]
Castanea	37	185	0	0	1.000	[0.91, 1.00]
Ceratonia	37	136	3	49	0.735	[0.58, 0.85]
Ebenus	6	30	0	0	1.000	[0.61, 1.00]
Erica	37	185	20	0	1.000	[0.91, 1.00]
Eucalyptus	37	165	4	20	0.892	[0.75, 0.96]
Ferula	30	145	0	5	0.967	[0.83, 0.99]
Myrtus	37	185	1	0	1.000	[0.91, 1.00]
Olea	37	185	27	0	1.000	[0.91, 1.00]
Origanum	37	169	12	16	0.914	[0.78, 0.97]
Oxalis	37	178	2	7	0.962	[0.85, 0.99]
Pinus	10	50	0	0	1.000	[0.72, 1.00]
Pistacia	12	54	1	6	0.900	[0.63, 0.98]
Salvia	37	185	5	0	1.000	[0.91, 1.00]
Satureja	25	106	0	19	0.848	[0.66, 0.94]
Sinapis	37	172	4	13	0.930	[0.80, 0.98]
Thymbra	37	177	28	8	0.957	[0.84, 0.99]
Urginea	37	179	6	6	0.968	[0.85, 0.99]
Vitis	37	180	36	5	0.973	[0.86, 1.00]

*Note:* TP, FP and FN are summed over five evaluations of the same test images (one per seed), so the totals are five times the class support. The confidence interval is computed for the mean recall with the number of distinct test images (n) as the sample size, because the five evaluations are not independent samples.

**Table 7:** Comparison of the YOLOv8s-cls, YOLO11s-cls and YOLO26s-cls baseline models on the unbalanced training set of Dataset 1 (test set, mean ± SD over five seeds).

Metrics	YOLOv8s-cls	YOLO11s-cls	YOLO26s-cls
Top-1 accuracy	0.9436 ± 0.0057	0.9507 ± 0.0079	0.9445 ± 0.0065
Top-5 accuracy	0.9941 ± 0.0019	0.9945 ± 0.0025	0.9948 ± 0.0021
Precision (macro)	0.9524 ± 0.0062	0.9570 ± 0.0069	0.9478 ± 0.0066
Precision (weighted)	0.9484 ± 0.0051	0.9527 ± 0.0078	0.9475 ± 0.0063
Recall (macro)	0.9471 ± 0.0053	0.9505 ± 0.0074	0.9460 ± 0.0072
Recall (weighted)	0.9436 ± 0.0057	0.9507 ± 0.0079	0.9445 ± 0.0065
F1-score (macro)	0.9469 ± 0.0062	0.9521 ± 0.0076	0.9451 ± 0.0065
F1-score (weighted)	0.9430 ± 0.0059	0.9503 ± 0.0081	0.9442 ± 0.0064

**Table 10:** Comparison of YOLOv8s-cls, YOLO11s-cls and YOLO26s-cls results on the unbalanced and balanced training sets (test set, mean ± SD over five seeds).

Metrics	YOLOv8s-cls (Unbalanced)	YOLOv8s-cls (Balanced)	YOLO11s-cls (Unbalanced)	YOLO11s-cls (Balanced)	YOLO26s-cls (Unbalanced)	YOLO26s-cls (Balanced)
Top-1 accuracy	0.9436 ± 0.0057	0.9618 ± 0.0034	0.9507 ± 0.0079	0.9586 ± 0.0038	0.9445 ± 0.0065	0.9586 ± 0.0088
Precision (macro)	0.9524 ± 0.0062	0.9677 ± 0.0032	0.9570 ± 0.0069	0.9654 ± 0.0048	0.9478 ± 0.0066	0.9594 ± 0.0090
Precision (weighted)	0.9484 ± 0.0051	0.9649 ± 0.0036	0.9527 ± 0.0078	0.9611 ± 0.0038	0.9475 ± 0.0063	0.9609 ± 0.0090
Recall (macro)	0.9471 ± 0.0053	0.9621 ± 0.0028	0.9505 ± 0.0074	0.9586 ± 0.0037	0.9460 ± 0.0072	0.9585 ± 0.0078
Recall (weighted)	0.9436 ± 0.0057	0.9618 ± 0.0034	0.9507 ± 0.0079	0.9586 ± 0.0038	0.9445 ± 0.0065	0.9586 ± 0.0088
F1-score (macro)	0.9469 ± 0.0062	0.9631 ± 0.0032	0.9521 ± 0.0076	0.9606 ± 0.0039	0.9451 ± 0.0065	0.9575 ± 0.0086
F1-score (weighted)	0.9430 ± 0.0059	0.9616 ± 0.0036	0.9503 ± 0.0081	0.9584 ± 0.0038	0.9442 ± 0.0064	0.9583 ± 0.0091

**Table 11:** Per-class F1-scores of the three architectures on the balanced training set.

Class	YOLO Models F1			Support
	YOLOv8s-cls	YOLOv11s-cls	YOLOv26s-cls	
Asphodelus	0.9532 ± 0.0320	0.9920 ± 0.0179	0.9310 ± 0.0299	12
Calicotome	0.9290 ± 0.0261	0.9307 ± 0.0097	0.9454 ± 0.0167	37
Castanea	1.0000 ± 0.0000	1.0000 ± 0.0000	1.0000 ± 0.0000	37
Ceratonia	0.8950 ± 0.0261	0.8997 ± 0.0275	0.9019 ± 0.0268	37
Ebenus	1.0000 ± 0.0000	1.0000 ± 0.0000	1.0000 ± 0.0000	6
Erica	0.9790 ± 0.0148	0.9763 ± 0.0107	0.9895 ± 0.0169	37
Eucalyptus	0.9527 ± 0.0164	0.9519 ± 0.0136	0.9670 ± 0.0186	37
Ferula	0.9831 ± 0.0000	0.9728 ± 0.0094	0.9831 ± 0.0000	30
Myrtus	0.9973 ± 0.0060	0.9894 ± 0.0110	0.9973 ± 0.0060	37
Olea	0.9516 ± 0.0238	0.9276 ± 0.0190	0.9318 ± 0.0245	37
Origanum	0.9407 ± 0.0161	0.9382 ± 0.0262	0.8927 ± 0.0468	37
Oxalis	1.0000 ± 0.0000	0.9784 ± 0.0118	0.9838 ± 0.0146	37
Pinus	1.0000 ± 0.0000	1.0000 ± 0.0000	1.0000 ± 0.0000	10
Pistacia	0.9186 ± 0.0212	0.8948 ± 0.0232	0.8790 ± 0.0211	12
Salvia	0.9893 ± 0.0060	0.9973 ± 0.0060	0.9920 ± 0.0073	37
Satureja	0.9633 ± 0.0171	0.9495 ± 0.0121	0.9173 ± 0.0145	25
Sinapis	0.9621 ± 0.0118	0.9569 ± 0.0173	0.9646 ± 0.0112	37
Thymbra	0.9504 ± 0.0054	0.9446 ± 0.0054	0.9314 ± 0.0283	37
Urginea	0.9891 ± 0.0061	0.9893 ± 0.0113	0.9891 ± 0.0178	37
Vitis	0.9082 ± 0.0109	0.9230 ± 0.0091	0.9534 ± 0.0242	37
**Macro F1**	**0.9631 ± 0.0032**	**0.9606 ± 0.0039**	**0.9575 ± 0.0086**	

**Table 12:** Macro F1-score and Ceratonia F1-score for the unbalanced, augmented–unbalanced and balanced training sets.

Metrics	YOLOv8s-cls	YOLO11s-cls	YOLO26s-cls
Macro F1			
YOLOv8s-cls	0.9469 ± 0.0062	0.9629 ± 0.0073	0.9631 ± 0.0032
YOLO11s-cls	0.9521 ± 0.0076	0.9547 ± 0.0144	0.9606 ± 0.0039
YOLO26s-cls	0.9451 ± 0.0065	0.9639 ± 0.0069	0.9575 ± 0.0086
Mean of three	0.9480	0.9605	0.9604
F1 (Ceratonia)			
YOLOv8s-cls	0.8381 ± 0.0401	0.8868 ± 0.0175	0.8950 ± 0.0261
YOLO11s-cls	0.8550 ± 0.0426	0.8726 ± 0.0370	0.8997 ± 0.0275
YOLO26s-cls	0.8758 ± 0.0244	0.9096 ± 0.0284	0.9019 ± 0.0268
Mean of three	0.8563	0.8897	0.8989

**Table 13:** Comparison of YOLOv8s-cls with cross-entropy (CE) and Focal Loss on the balanced training set.

Configuration	Val. Macro F1	Test Accuracy	Test Macro F1	F1 (Ceratonia)	F1 (Vitis)
CE	0.9821 ± 0.0012	0.9618 ± 0.0034	0.9631 ± 0.0032	0.8950 ± 0.0261	0.9082 ± 0.0109
Focal, γ = 0.5 (selected)	0.9813 ± 0.0017	0.9556 ± 0.0042	0.9567 ± 0.0043	0.8665 ± 0.0139	0.8916 ± 0.0109
Focal, γ = 1.0	0.9795 ± 0.0024	0.9560 ± 0.0094	0.9577 ± 0.0099	0.8932 ± 0.0255	0.9134 ± 0.0220
Focal, γ = 2.0	0.9804 ± 0.0015	0.9579 ± 0.0069	0.9593 ± 0.0075	0.8750 ± 0.0218	0.9077 ± 0.0170

**Table 14:** Per-class F1-scores of YOLOv8s-cls with CE and Focal Loss on the balanced training set.

Class	F1 (CE)	F1 (Focal, γ = 2.0)	F1 (Focal, γ = 1.0)	F1 (Focal, γ = 2.0 + α-Boost)	SE-Block
Asphodelus	0.9532 ± 0.0320	0.9366 ± 0.0355	0.9520 ± 0.0438	0.9378 ± 0.0580	0.9400 ± 0.0537
Calicotome	0.9290 ± 0.0261	0.9284 ± 0.0216	0.9256 ± 0.0081	0.9343 ± 0.0286	0.9396 ± 0.0123
Castanea	1.0000 ± 0.0000	0.9947 ± 0.0073	0.9947 ± 0.0073	0.9947 ± 0.0073	0.9947 ± 0.0118
Ceratonia	0.8950 ± 0.0261	0.8665 ± 0.0139	0.8932 ± 0.0255	0.8750 ± 0.0218	0.8920 ± 0.0184
Ebenus	1.0000 ± 0.0000	1.0000 ± 0.0000	1.0000 ± 0.0000	1.0000 ± 0.0000	1.0000 ± 0.0000
Erica	0.9790 ± 0.0148	0.9843 ± 0.0170	0.9763 ± 0.0107	0.9841 ± 0.0110	0.9894 ± 0.0110
Eucalyptus	0.9527 ± 0.0164	0.9555 ± 0.0062	0.9556 ± 0.0152	0.9635 ± 0.0079	0.9641 ± 0.0160
Ferula	0.9831 ± 0.0000	0.9763 ± 0.0093	0.9728 ± 0.0152	0.9763 ± 0.0093	0.9795 ± 0.0078
Myrtus	0.9973 ± 0.0060	0.9973 ± 0.0060	0.9973 ± 0.0060	0.9973 ± 0.0060	0.9973 ± 0.0060
Olea	0.9516 ± 0.0238	0.9416 ± 0.0137	0.9514 ± 0.0180	0.9418 ± 0.0203	0.9488 ± 0.0086
Origanum	0.9407 ± 0.0161	0.9206 ± 0.0227	0.9200 ± 0.0298	0.9254 ± 0.0289	0.9238 ± 0.0088
Oxalis	1.0000 ± 0.0000	0.9973 ± 0.0060	0.9973 ± 0.0061	0.9946 ± 0.0074	0.9944 ± 0.0124
Pinus	1.0000 ± 0.0000	1.0000 ± 0.0000	1.0000 ± 0.0000	1.0000 ± 0.0000	1.0000 ± 0.0000
Pistacia	0.9186 ± 0.0212	0.9012 ± 0.0177	0.9186 ± 0.0212	0.9281 ± 0.0260	0.9106 ± 0.0034
Salvia	0.9893 ± 0.0060	0.9947 ± 0.0073	0.9894 ± 0.0110	0.9815 ± 0.0071	0.9947 ± 0.0073
Satureja	0.9633 ± 0.0171	0.9629 ± 0.0093	0.9365 ± 0.0161	0.9539 ± 0.0099	0.9454 ± 0.0200
Sinapis	0.9621 ± 0.0118	0.9570 ± 0.0145	0.9567 ± 0.0177	0.9595 ± 0.0166	0.9623 ± 0.0106
Thymbra	0.9504 ± 0.0054	0.9360 ± 0.0209	0.9143 ± 0.0267	0.9340 ± 0.0051	0.9313 ± 0.0144
Urginea	0.9891 ± 0.0061	0.9918 ± 0.0075	0.9890 ± 0.0116	0.9973 ± 0.0061	0.9918 ± 0.0124
Vitis	0.9082 ± 0.0109	0.8916 ± 0.0109	0.9134 ± 0.0220	0.9077 ± 0.0170	0.9008 ± 0.0236
**Macro F1**	0.9631 ± 0.0032	0.9567 ± 0.0043	0.9577 ± 0.0099	0.9593 ± 0.0075	0.9600 ± 0.0048
**Weighted F1**	0.9616 ± 0.0036	0.9554 ± 0.0042	0.9558 ± 0.0094	0.9576 ± 0.0069	0.9591 ± 0.0039
Ceratonia P/R	1.000/0.811	0.987/0.773	0.987/0.816	1.000/0.778	1.000/0.805

**Table 15:** Results of CE, Focal Loss and SE-Block for the three YOLO architectures on the balanced training set.

Architecture	Configuration	Val. Macro F1	Test Top-1 Acc.	Test Macro F1	F1 (Ceratonia)	F1 (Vitis)
YOLOv8s-cls	CE	0.9821 ± 0.0012	0.9618 ± 0.0034	0.9631 ± 0.0032	0.8950 ± 0.0261	0.9082 ± 0.0109
YOLOv8s-cls	Focal (γ = 0.5)	0.9813 ± 0.0017	0.9556 ± 0.0042	0.9567 ± 0.0043	0.8665 ± 0.0139	0.8916 ± 0.0109
YOLOv8s-cls	SE-Block	0.9821 ± 0.0024	0.9592 ± 0.0038	0.9600 ± 0.0048	0.8920 ± 0.0184	0.9008 ± 0.0236
YOLO11s-cls	CE	0.9775 ± 0.0039	0.9586 ± 0.0037	0.9606 ± 0.0039	0.8997 ± 0.0275	0.9230 ± 0.0091
YOLO11s-cls	Focal (γ = 1.0)	0.9784 ± 0.0025	0.9615 ± 0.0075	0.9638 ± 0.0071	0.9006 ± 0.0447	0.9325 ± 0.0324
YOLO11s-cls	SE-Block	0.9759 ± 0.0036	0.9631 ± 0.0056	0.9644 ± 0.0044	0.9084 ± 0.0377	0.9484 ± 0.0233
YOLO26s-cls	CE	0.9797 ± 0.0032	0.9586 ± 0.0088	0.9575 ± 0.0086	0.9019 ± 0.0268	0.9534 ± 0.0242
YOLO26s-cls	Focal (γ = 0.5)	0.9817 ± 0.0028	0.9631 ± 0.0039	0.9612 ± 0.0044	0.9312 ± 0.0133	0.9683 ± 0.0199
YOLO26s-cls	SE-Block	0.9803 ± 0.0078	0.9638 ± 0.0086	0.9611 ± 0.0093	0.9316 ± 0.0198	0.9605 ± 0.0126

**Table 16:** Differences from CE for Focal Loss and SE-Block across the three architectures.

Architecture	Method	Δ Macro F1	*p*	Holm *p*	Δ F1 (Ceratonia)	*p*	Holm *p*
YOLOv8s-cls	Focal (γ = 0.5)	−0.0064	0.029	0.35	−0.0285	0.074	0.79
YOLOv8s-cls	SE-Block	−0.0031	0.27	1.00	−0.0031	0.84	1.00
YOLO11s-cls	Focal (γ = 1.0)	+0.0032	0.42	1.00	+0.0009	0.97	1.00
YOLO11s-cls	SE-Block	+0.0038	0.19	1.00	+0.0087	0.69	1.00
YOLO26s-cls	Focal (γ = 0.5)	+0.0037	0.43	1.00	+0.0293	0.072	0.79
YOLO26s-cls	SE-Block	+0.0035	0.55	1.00	+0.0296	0.085	0.79

**Table 17:** Results of the reproduced RCANet compared with YOLOv8s-cls and EfficientNet-B3 on Dataset 1 (balanced training set) and Dataset 2.

Model	D1: Macro F1	D1: Weighted F1	D1: F1 (Ceratonia)	D2: Macro F1	D2: Weighted F1	D2: F1 (Ceratonia)
RCANet-84 (original setting)	0.9560 ± 0.0074	0.9526 ± 0.0075	0.8505 ± 0.0240	0.9165 ± 0.0054	0.9184 ± 0.0064	0.6975 ± 0.0411
RCANet-224	0.9621 ± 0.0059	0.9594 ± 0.0068	0.8550 ± 0.0208	0.9502 ± 0.0078	0.9512 ± 0.0072	0.7393 ± 0.0991
YOLOv8s-cls (CE)	0.9631 ± 0.0032	0.9616 ± 0.0036	0.8950 ± 0.0261	0.9416 ± 0.0092	0.9436 ± 0.0069	0.7351 ± 0.0401
EfficientNet-B3	0.9653 ± 0.0079	0.9630 ± 0.0059	0.8448 ± 0.0301	0.9658 ± 0.0083	0.9646 ± 0.0083	0.8247 ± 0.0335

*Note:* D1: Dataset 1, balanced training set (test n = 613); D2: Dataset 2, original (unbalanced) training split (test n = 1345). Mean ± SD over five seeds.

**Table 18:** Per-class results of EfficientNet-B3 on the unbalanced and balanced training sets of Dataset 1 and on Dataset 2.

Class	Dataset 1			Dataset 2	
	Unbalanced (P/R/F1)	Balanced (P/R/F1/S)	Support	(P/R/F1)	Support
Asphodelus	0.983/0.933/0.957	1.000/0.900/0.945	12	1.000/1.000/1.000	17
Calicotome	0.930/0.968/0.948	0.960/0.973/0.966	37	0.949/0.994/0.971	100
Castanea	0.984/1.000/0.992	1.000/1.000/1.000	37	0.989/1.000/0.994	70
Ceratonia	0.986/0.697/0.815	0.988/0.741/0.845	37	0.884/0.776/0.825	50
Ebenus	1.000/1.000/1.000	1.000/1.000/1.000	6	1.000/1.000/1.000	11
Erica	0.959/1.000/0.979	0.974/1.000/0.987	37	0.946/1.000/0.972	70
Eucalyptus	0.967/0.946/0.956	0.953/0.962/0.957	37	0.981/0.891/0.933	70
Ferula	0.949/0.967/0.958	0.993/0.967/0.980	30	0.963/0.976/0.969	42
Myrtus	0.995/1.000/0.997	0.995/1.000/0.997	37	0.996/0.998/0.997	157
Olea	0.853/1.000/0.920	0.869/1.000/0.930	37	0.945/0.991/0.968	158
Origanum	0.944/0.973/0.958	0.937/0.968/0.952	37	0.977/0.949/0.962	70
Oxalis	1.000/0.984/0.992	0.984/0.989/0.987	37	0.992/1.000/0.996	70
Pinus	1.000/1.000/1.000	1.000/1.000/1.000	10	1.000/0.987/0.993	15
Pistacia	0.951/0.950/0.950	0.968/0.967/0.967	12	0.937/1.000/0.967	17
Salvia	0.975/1.000/0.987	0.990/1.000/0.995	37	0.970/0.997/0.983	70
Satureja	0.991/0.864/0.923	1.000/0.896/0.945	25	0.983/0.960/0.971	35
Sinapis	0.925/0.951/0.937	0.979/0.957/0.967	37	0.964/0.958/0.961	100
Thymbra	0.979/0.935/0.956	0.949/0.989/0.968	37	0.991/0.896/0.941	73
Urginea	1.000/0.973/0.986	1.000/0.973/0.986	37	0.988/0.980/0.984	50
Vitis	0.870/0.962/0.913	0.885/0.989/0.934	37	0.924/0.936/0.929	100
Macro avg	0.962/0.955/0.956	0.971/0.964/0.965	613	0.969/0.964/0.966	1345
Weighted avg	0.958/0.954/0.953	0.967/0.964/0.963	613	0.966/0.965/0.965	1345
Accuracy	*0.9537 ± 0.0059*	*0.9638 ± 0.0055*		*0.9652 ± 0.0080*	
Macro F1	*0.9561 ± 0.0064*	*0.9653 ± 0.0079*		*0.9658 ± 0.0082*	

**Table 19:** Comparison of EfficientNet-B3 with the YOLO baseline models on the unbalanced training set of Dataset 1.

Metrics	YOLOv8s-cls	YOLO11s-cls	YOLO26s-cls	EfficientNet-B3
Top-1 accuracy	0.9436 ± 0.0057	0.9507 ± 0.0079	0.9445 ± 0.0065	0.9537 ± 0.0059
F1-score (macro)	0.9469 ± 0.0062	0.9521 ± 0.0076	0.9451 ± 0.0065	0.9561 ± 0.0064
F1-score (weighted)	0.9430 ± 0.0059	0.9503 ± 0.0081	0.9442 ± 0.0064	0.9527 ± 0.0059
F1 (Ceratonia)	0.8381 ± 0.0401	0.8550 ± 0.0426	0.8758 ± 0.0244	0.8148 ± 0.0415

**Table 20:** Macro F1-score and Ceratonia F1-score of all configurations trained on the balanced training set of Dataset 1.

Configuration	F1-Score (Macro)	F1 (Ceratonia)
YOLOv8s-cls (CE)	0.9631 ± 0.0032	0.8950 ± 0.0261
YOLOv8s-cls (Focal, γ = 0.5)	0.9567 ± 0.0043	0.8665 ± 0.0139
YOLOv8s-cls (SE-Block)	0.9600 ± 0.0048	0.8920 ± 0.0184
YOLO11s-cls (CE)	0.9606 ± 0.0039	0.8997 ± 0.0275
YOLO11s-cls (Focal, γ = 1.0)	0.9638 ± 0.0071	0.9006 ± 0.0447
YOLO11s-cls (SE-Block)	0.9644 ± 0.0044	0.9084 ± 0.0377
YOLO26s-cls (CE)	0.9575 ± 0.0086	0.9019 ± 0.0268
YOLO26s-cls (Focal, γ = 0.5)	0.9612 ± 0.0044	0.9312 ± 0.0133
YOLO26s-cls (SE-Block)	0.9611 ± 0.0093	0.9316 ± 0.0198
RCANet-224	0.9621 ± 0.0059	0.8550 ± 0.0208
EfficientNet-B3	0.9653 ± 0.0079	0.8448 ± 0.0301

**Table 21:** Comparison of the results of this study with results published for CPD-1.

Source	Method	Evaluation	Reported/Obtained Result
Tsiknakis et al. [10]	Soft-voting ensemble of four pretrained CNNs	single hold-out split (50% test), one run	Accuracy = 97.52%, macro F1 = 96.89%, weighted F1 = 97.53%
Mahmood et al. [11]	APFA-Net (feature aggregation with SE-Blocks), trained from scratch	cross-validation	Accuracy = 98.33%, F1 = 98.39%
Zolfaghari and Sajedi [12]	RCANet (ResNet18 + VDAB)	5-fold cross-validation	Weighted accuracy = 98.71%, weighted F1 = 98.69%
This work (reproduction of [11])	RCANet-224	Dataset 1/Dataset 2, 5 seeds	Weighted F1 = 95.94 ± 0.68%/95.12 ± 0.72%
This work	EfficientNet-B3 (best single model)	Dataset 1, balanced, 5 seeds	Accuracy = 96.38 ± 0.55%, macro F1 = 96.53 ± 0.79%, weighted F1 = 96.30 ± 0.59%
This work	Ensemble selected on the validation set	Dataset 1, balanced, 5 seeds	Accuracy = 97.26 ± 0.27%, macro F1 = 97.20 ± 0.28%, weighted F1 = 97.24 ± 0.28%

**Table 22:** Results of the baseline models on Dataset 2.

Metrics	YOLOv8s-cls	YOLO11s-cls	YOLO26s-cls	EfficientNet-B3	RCANet-224
Top-1 accuracy	0.9447 ± 0.0068	0.9501 ± 0.0077	0.9497 ± 0.0088	0.9652 ± 0.0080	0.9527 ± 0.0063
F1-score (macro)	0.9416 ± 0.0092	0.9472 ± 0.0079	0.9459 ± 0.0097	0.9658 ± 0.0082	0.9502 ± 0.0078
F1-score (weighted)	0.9436 ± 0.0069	0.9497 ± 0.0079	0.9494 ± 0.0089	0.9647 ± 0.0083	0.9512 ± 0.0072
F1 (Ceratonia)	0.7351 ± 0.0401	0.8173 ± 0.0557	0.8005 ± 0.0271	0.8247 ± 0.0335	0.7393 ± 0.0991

**Table 23:** F1-score of the Ceratonia class on Dataset 1 and Dataset 2 (unbalanced training in both cases).

Architecture	F1 (Dataset 1)	F1 (Dataset 2)	ΔF1	*p* (Holm)
YOLOv8s-cls	0.8381 ± 0.0401	0.7351 ± 0.0401	−0.1030	0.011
YOLO11s-cls	0.8550 ± 0.0426	0.8173 ± 0.0557	−0.0377	0.53
YOLO26s-cls	0.8758 ± 0.0244	0.8005 ± 0.0271	−0.0753	0.007
EfficientNet-B3	0.8148 ± 0.0415	0.8247 ± 0.0335	+0.0099	0.69

**Table 24:** Results of single models and ensembles on the balanced training set of Dataset 1.

Model/Selection	Accuracy	Macro F1	Weighted F1	F1 (Ceratonia)	F1 (Vitis)
Best single model, selected on validation (YOLOv8s-cls, CE)	0.9618 ± 0.0034	0.9631 ± 0.0032	0.9616 ± 0.0036	0.8950 ± 0.0261	0.9082 ± 0.0109
Single model with highest test mean (EfficientNet-B3) *	0.9638 ± 0.0055	0.9653 ± 0.0079	0.9630 ± 0.0059	0.8448 ± 0.0301	0.9337 ± 0.0051
**Ensemble, protocol A (validation, one configuration)**	**0.9726 ± 0.0027**	**0.9720 ± 0.0028**	**0.9724 ± 0.0028**	0.9047 ± 0.0292	0.9488 ± 0.0086
Ensemble, protocol B (validation, per seed)	–	0.9745 ± 0.0022	–	–	–
Ensemble, test selection (reference only) *	–	0.9786 ± 0.0013	–	–	–

* Identified using test-set results; reported for reference only.

**Table 25:** Comparison of mixed (EfficientNet-B3 + YOLO) and YOLO-only ensembles (mean over all configurations in each group and over five seeds).

Ensemble Composition	Number of Configurations	Val. Macro F1	Test Macro F1	F1 (Ceratonia)	F1 (Vitis)
EfficientNet-B3 + YOLO	45	0.9846	0.9731	0.905	0.954
YOLO only	33	0.9810	0.9670	0.916	0.945
Difference (*p*, paired over seeds)		+0.0036 (0.0001)	+0.0061 (0.0009)	−0.011 (0.10)	+0.009 (0.07)

**Table 26:** Ablation study of Focal Loss and SE-Block in YOLOv8s-cls on the balanced training set.

Configuration	Macro F1	F1 (Ceratonia)	F1 (Vitis)
CE	0.9631 ± 0.0032	0.8950 ± 0.0261	0.9082 ± 0.0109
Focal Loss (γ = 0.5)	0.9567 ± 0.0043	0.8665 ± 0.0139	0.8916 ± 0.0109
SE-Block	0.9600 ± 0.0048	0.8920 ± 0.0184	0.9008 ± 0.0236
Focal Loss + SE-Block	0.9600 ± 0.0055	0.8817 ± 0.0252	0.9060 ± 0.0180

**Table 27:** Misclassifications of Ceratonia test images (balanced training set of Dataset 1, summed over five seeds).

Model	Recall	Predicted as Vitis	Olea	Origanum	Eucalyptus	Sinapis or Salvia	Other	Other Classes Predicted as Ceratonia
YOLOv8s-cls	150/185 (0.811)	22	5	2	4	1	1	0
YOLO11s-cls	153/185 (0.827)	13	10	6	0	2	1	2
YOLO26s-cls	157/185 (0.849)	10	10	4	0	2	2	6
EfficientNet-B3	137/185 (0.741)	19	14	7	5	2	1	2
RCANet-224	139/185 (0.751)	22	10	6	4	0	4	1
**Total**	**736/925**	**86 (46%)**	**49 (26%)**	**25 (13%)**	**13 (7%)**	**7 (4%)**	**9 (5%)**	**11**

**Table 28:** Effect of the number of original Ceratonia training images (YOLOv8s-cls; each set augmented to 716 Ceratonia images).

Original Ceratonia Images	F1 (Ceratonia)	F1 (Vitis)	Macro F1	Accuracy
26	0.8777 ± 0.0404	0.9118 ± 0.0228	0.9567 ± 0.0048	0.9547 ± 0.0045
18	0.8132 ± 0.0347	0.8982 ± 0.0056	0.9517 ± 0.0038	0.9504 ± 0.0038
10	0.7778 ± 0.0407	0.8647 ± 0.0368	0.9475 ± 0.0030	0.9462 ± 0.0049

## Data Availability

The raw data supporting the conclusions of this article will be made available by the authors on request.

## Abbreviations

The following abbreviations are used in this manuscript:

AdamW	Adam optimizer with decoupled weight decay
APFA-Net	Attention-guided Pollen Features Aggregation Network
AUC	Area under the receiver operating characteristic curve
CBAM	Convolutional Block Attention Module
CE	Cross-entropy
CI	Confidence interval
CNN	Convolutional neural network
CPD-1	Cretan Pollen Dataset v1
CUDA	Compute Unified Device Architecture
FN	False negative
FP	False positive
GPU	Graphics processing unit
Grad-CAM	Gradient-weighted Class Activation Mapping
PDO	Protected Designation of Origin
pp	Percentage points
RCANet	[tekshirish kerak, quyiga qarang]
ReLU	Rectified linear unit
SD	Standard deviation
SE	Squeeze-and-Excitation
SENet	Squeeze-and-Excitation Network
SGD	Stochastic gradient descent
TP	True positive
VDAB	Ventral–Dorsal Attention Block
YOLO	You Only Look Once
YOLO-cls	Classification variant of YOLO
